# Calcium Carbonate and Water Pyrolysis Measurements Suggest Minor Adjustment to the VPDB and VSMOW‐SLAP δ^18^O Scale Relation

**DOI:** 10.1002/rcm.10093

**Published:** 2025-06-17

**Authors:** Anita Th. Aerts‐Bijma, Dipayan Paul, Albert C. van Buuren, Vivian R. Kroon, Harro A. J. Meijer

**Affiliations:** ^1^ Centre for Isotope Research (CIO), Energy and Sustainability Research Institute Groningen University of Groningen Groningen The Netherlands

**Keywords:** ^18^O scales, ^18^O VPDB‐VSMOW relation, calcite ^18^O reference material, EA‐pyrolysis‐IRMS

## Abstract

**Rationale:**

Measurements of the oxygen isotopic composition of materials are widely used in many fields. These oxygen isotopic compositions are expressed using different scales. These scales are only linked indirectly, because water and calcium carbonate reference materials, used to establish these oxygen isotope delta scales, have to be converted to CO_2_ first, and the isotopic fractionation of these conversions has only been measured a few times in the past. The anchoring of the two oxygen isotope delta scales is therefore currently suboptimal.

**Methods:**

Primary reference materials, both waters and calcium carbonates, were pyrolyzed within a single measurement sequence in a high‐temperature elemental analyzer–pyrolysis system connected to an isotope ratio mass spectrometer in continuous‐flow mode. As calcium carbonate is difficult to pyrolyze completely, additives were added to reach a 100% yield. The *δ* ^18^O of the calcium carbonates were normalized on the VSMOW‐SLAP scale using VSMOW2 and in‐house water references.

**Results:**

The average results of 6 international calcites references measured in five independent sequences of pyrolysis measurements showed a difference of 0.07 to 0.09‰ with the presently described relationship in literature between the *δ* ^18^O VPDB and *δ* ^18^O VSMOW‐SLAP scale.

**Conclusions:**

The outcome of the study made the direct comparison of the two different ^18^O scales possible. Our results demonstrate a small discrepancy in the presently recommended relation between the two oxygen isotope delta scales.

## 1 Introduction

Isotope ratio mass spectrometers are commonly used to measure isotopic signals. Instead of measuring these small isotopic variations directly, the isotopic differences are compared to the isotopic compositions of reference materials (RMs). Therefore, isotopic scales are defined by RMs. To ensure that measurements remain comparable and consistent worldwide, ideally the RM measurements would be traceable to the standard measurement system, SI (Système Internationale). This traceability can be established by means of an unbroken chain of calibrations or comparisons linking them to relevant primary standards of the SI units of measurement (coordinated by the International Bureau of Weights and Measures, BIPM). Currently, however, the SI traceability of many isotope measurements is not ensured. Work in this study tries to contribute to this traceability and the consistency in isotopic data.

Oxygen isotope delta values (*δ* ^18^O) are widely used in various fields due to their ability to provide insights into environmental, geological, and biological processes. To express oxygen isotopic compositions of calcium carbonate minerals, the VPDB (Vienna Peedee belemnite) ^18^O scale is used [1].

For oxygen isotope measurements in water, but also for other types of samples, such as oxides and silicates, another oxygen isotope scale is used, the *δ* ^18^O VSMOW (Vienna Standard Mean Ocean Water) scale [2].

The relation between the two different *δ* ^18^O scales is based on historical data, often without mentioning the uncertainty of these data, and the relation is not necessarily accurate.

An accurate link between the two ^18^O scales would be beneficial to for example the metrological community, being able to combine *δ* ^18^O data gathered from both water and calcium carbonate samples in a reliable way.

Currently the link between the two *δ* ^18^O scales is established through a conversion of the water and calcium carbonate RMs to carbon dioxide, because the measurand of isotope ratio mass spectrometers is gas. These conversion reactions of the water and calcium carbonate RMs to CO_2_ result in isotopic fractionation. This fractionation leads to an extra uncertainty in the link between the two *δ* ^18^O scales. In this study, water and calcium carbonate RMs are pyrolyzed within the same measurement sequence, such that the results of both RMs at the same reaction conditions can be directly compared. Direct anchoring of the two ^18^O scales, avoiding the conversion to CO_2_ first, will make the whole scheme of conversion equations much stronger. By doing so, this study contributes to the metrological traceability of the ^18^O scales to the SI system.

Ideally, an isotopic scale is realized using two different RMs with a large difference in their isotopic value. Using a two‐point‐calibration scale will improve the accuracy and the interlaboratory agreement of isotopic measurements.

The primary reference for the VSMOW ^18^O scale is VSMOW, SLAP (Standard Light Antarctic Precipitation) was introduced as a second reference material to provide a two‐point calibration for the water scale [2, 3]. VSMOW and SLAP are no longer available from the IAEA, and although original VSMOW might still be available from other sources (such as the Reston Stable Isotope Laboratory (RSIL)), VSMOW2 and SLAP2 are now the recommended RMs to realize the *δ* ^18^O_VSMOW‐SLAP_ scale with the lowest uncertainty, and these are called secondary RMs [4]. The recommended and defined *δ* ^18^O_VSMOW‐SLAP_ values for VSMOW2 and SLAP2 are 0.00 ± 0.02‰ and −55.50 ± 0.02‰ respectively [5].

The primary reference for the VPDB ^18^O scale is NBS 19, which is not freely available at the IAEA anymore, although 1 kg of NBS 19 has been quarantined [6]. Again, although NBS 19 is still available from RSIL for calibration of secondary isotopic reference materials, the currently recommended secondary reference for the VPDB ^18^O scale is IAEA‐603, a low‐magnesium marble calcite, [4, 7]. Nishida and Ishimura [8] found some (1–2 grains per 100) with lower δ^13^C and δ^18^O values, which is important for analysis of small amounts (smaller than 120 μg). In our case, with 0.3 mg samples, this effect is considered negligible (if would be less than 0.004‰). Unfortunately, there is no formal consensus about the second anchor of the VPDB scale. NBS 18, a carbonatite [9] has been recommended (but never officially designated) as a second anchor. This RM is known to definitely suffer from isotopic inhomogeneity at the grain size level [10]. The recommended assigned *δ* ^18^O_VPDB_ values and their associated uncertainties for IAEA‐603 and NBS 18 are −2.37 ± 0.04‰ and −23.01 ± 0.03‰, respectively. [5].

Unlike the *δ* ^18^O values for NBS 19, VSMOW and SLAP, which are the primary RMs of the two ^18^O scales that were defined with zero uncertainty, the *δ* ^18^O values for the secondary RMs IAEA‐603, VSMOW2 and SLAP2 include uncertainties estimated in a quantifiable manner. [5].

As mentioned before, to measure *δ* ^18^O values with isotope ratio mass spectrometry (IRMS) in carbonate minerals or in water samples, the materials need to be converted to carbon dioxide gas first, because the measurand in IRMS is carbon dioxide gas. This conversion means that the ^18^O scales actually are VSMOW‐SLAP‐CO_2_ and VPDB‐CO_2_ scales. Calcium carbonate samples are converted to CO_2_ by an acid digestion reaction [11] under specific reaction conditions. Water samples are converted to CO_2_ by an equilibration reaction with CO_2_ gas of known isotopic ratios under well‐defined conditions [12].

Both reactions result in isotopic fractionation, meaning that *δ* ^18^O of the original calcium carbonate material and *δ* ^18^O of the original water, are not the same as *δ* ^18^O of the formed carbon dioxide. This change in isotopic composition is quantified by a fractionation factor α [13]. The fractionation factor is unique to certain chemical reactions under particular reaction conditions. For the reaction from calcite to carbon dioxide under specific circumstances (at 25 °C), the commonly accepted value for ^18^ α=1.01025 (quoted by Friedman and O'Neil in [14]). For the equilibration reaction between water and carbon dioxide, at well‐defined conditions (at 25 °C), the commonly accepted value for ^18^ α=1.0412 (set by O'Neil in [15]).

**FIGURE 1:**
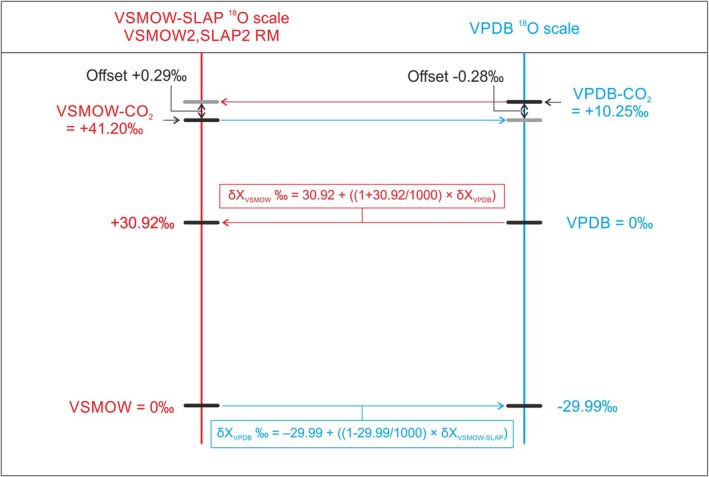
The *δ* ^18^O isotope scales based on water‐CO_2_ equilibration (VSMOW‐SLAP, left, in red) and based on the calcite‐phosphoric acid reaction (VPDB, right, in blue). Of all the numbers indicated, the two so‐called zero points, are per definition, while the isotopic fractionations VSMOW‐SLAP to VSMOW‐SLAP‐CO_2_ and VPDB to VPDB‐CO_2_ (41.20‰ and 10.25‰) are the commonly accepted values, based on measurements from decades ago. The offsets can in principle be directly measured. Finally, the value −29.99‰ for VPDB on the VSMOW‐SLAP scale, and inversely the value +30.92‰ of VSMOW‐SLAP on the VPDB scale, have been merely computed from the other values. [5].

A laboratory that is capable of realizing both ^18^O‐CO_2_ scales can measure the difference in *δ* ^18^O of both produced CO_2_’s [5, 16, 17]. The measurements of these offsets (0.28–0.29‰, see Figure 1), together with the fractionation factors, provide the currently described link between the two ^18^O scales. Per definition, the *δ* ^18^O value for VPDB is 0 on the VPDB ^18^O scale and that for VSMOW is 0 on the VSMOW‐SLAP ^18^O scale. Using the two fractionation factors, and the measured offset, one can compute the conversion values between the zero points of the two ^18^O scales: 0 on the VSMOW‐SLAP scale is +30.92‰ on the VPDB scale and 0 on the VPDB scale is −29.99‰ on the VSMOW‐SLAP scale (first cited by [18], finally established as commonly accepted values in [6, 19]), leading to the following conversion equations from one ^18^O scale to the other for an unknown sample x:

(1) δO18XVSMOW−SLAP=30.92+1+30.921000*δO18XVPDB‰

(2) δO18XVPDB=−29.99+1+−29.991000*δO18XVSMOW−SLAP‰

The scale conversion points +30.92‰ and −29.99‰ are called zero‐points throughout this manuscript. In the Supporting Information (at the end of this article) the derivation of Equations 1 and 2 is shown as well as the fact that Equations 1 and 2 are in full accord with each other.

Figure 1 is the most recent diagram that has been published and shows the commonly accepted points on the two scales and their conversions based on the currently recommended values. The fractionation factors mentioned before, 1.01025 and 1.0412 are displayed as 10.25‰ and 41.2‰ isotopic fractionation from VPDB to VPDB‐CO_2_ and VSMOW‐SLAP to VSMOW‐SLAP‐CO_2_, respectively, such that *δ* ^18^O SLAP = −55.5‰ [5].

Great strides have already been made to connect the *δ* ^18^O VSMOW‐SLAP scale to the SI. The combination of the experimental determination of the absolute ^18^O abundance of VSMOW [20] and the determination of the quantitative difference in *δ* ^18^O between VSMOW and SLAP [21] provide this SI traceability. However, this metrological traceability does not apply to the *δ* ^18^O VPDB scale. Unlike the ^18^O water scale, the ^18^O scale of the calcium carbonates is an artefact‐based scale only, or in other words the *δ* ^18^O VPDB scale is based on RMs and is not connected to the SI system. Moreover, the reproduction of the ^18^O scale by the acid digestion reaction is intrinsically error‐prone. By linking the water and the calcites^18^O scale directly to each other, meaning avoiding the extra step of the conversion to carbon dioxide first, the *δ* ^18^O VPDB scale would, in fact, become SI traceable as well.

Prior to these pyrolysis experiments, other possible oxygen sources in the calcium carbonate RMs were investigated, as the pyrolysis process, contrary to the calcite‐phosphoric acid reaction, is not selective for calcium carbonate only; it will decompose other oxygen‐bearing molecules as well. Therefore, qualitative analysis of the composition of calcium carbonate RMs was performed to gain insights in the purity of the RMs. Besides the composition of the RMs, the crystal structure of the calcium carbonate RMs was also explored. Natural calcium carbonate can exist in multiple phases such as calcite, aragonite, and dolomite, and each structure has different physical and chemical properties, thus possibly impacting complete pyrolysis. The crystal structure of the carbonates is also important for another reason: the isotopic fractionation of the phosphoric acid reaction with carbonates shows a dependence on the type of carbonate [19]. Therefore, in this study, reference materials predominantly characterized as calcites were chosen.

## 2 Materials and Methods

The experiments for this study were aimed at linking the *δ* ^18^O VPDB scale to the *δ* ^18^O VSMOW‐SLAP scale through the pyrolysis of calcite reference materials and water reference materials within the same measurement sequence.

Several international RMs, instruments, and procedures were used, which are described in this section.

### 2.1 Reference Materials RMs

#### 2.1.1 Calcite RMs

Table 1 shows an overview of the international calcium carbonate RMs that were selected for this study. The assigned *δ* ^18^O values and their associated uncertainties from literature, are all on the *δ* ^18^O VPDB scale. Conversions from *δ* ^18^O_VPDB_ values to *δ* ^18^O_VSMOW‐SLAP_ values were calculated by using the currently recommended conversion relation [1] mentioned in the introduction and derived in the Supporting Information.

**TABLE 1:** The *δ* ^18^O (VPDB) assigned values and calculated *δ* ^18^O (VSMOW‐SLAP) values of selected international calcium carbonate reference materials.

Reference material	Unit ID^a^	*δ* ^18^O_VPDB_ (‰)	*δ* ^18^O_VSMOW‐SLAP_ (‰) calculated
NBS 19	—	−2.2^b^	28.65
NBS 18	1079	−23.01 ± 0.03	7.20
IAEA‐603	1007	−2.37 ± 0.04	28.48
IAEA‐610	102	−18.834 ± 0.044	11.50
IAEA‐611	188	−4.224 ± 0.048	26.57
IAEA‐612	135	−12.079 ± 0.062	18.47
USGS44^c^	—	−15.66 ± 0.02 (CIO value)	14.78
USGS44^c^	—	−15.75 ± 0.02 (BGC value)	14.68

^a^ Ampoule unit identification number.

^b^ The primary RM NBS 19 does not have an uncertainty, but IAEA‐603, the recommended secondary RM for the realization of the ^18^O VPDB scale with the lowest uncertainty, does.

^c^ BGC‐lab and CIO found different *δ* ^18^O values for USGS44 calcium carbonate [22].

#### 2.1.2 Water RMs

In addition to the calcite RMs, water RMs were used that have established values on the *δ* ^18^O VSMOW‐SLAP scale. The secondary water RMs for the VSMOW‐SLAP scale are VSMOW2 and SLAP2. Their measured *δ* ^18^O values are 0.00 ± 0.02‰ and −55.50 ± 0.02‰, respectively. As all the calcite RMs have *δ* ^18^O_VSMOW‐SLAP_ values between 0 and +30‰ (see Table 1), additional water RMs were prepared from mixtures of IAEA‐607 (*δ* ^18^O_VSMOW‐SLAP_ = 99.02 ± 0.13‰, [23]) and USGS46 (*δ* ^18^O_VSMOW‐SLAP_ = −29.80 ± 0.03 ‰, [24]), such that the whole range of *δ* ^18^O_VSMOW‐SLAP_ of the calcites was covered. The prepared water mixtures were called sample 1 to sample 3. As measuring small differences in *δ* ^18^O values is more precise than measuring larger differences, sample 1 was prepared close to *δ* ^18^O _IAEA‐603_ and sample 2 was prepared close to *δ* ^18^O_USGS44_. To complement a broad range of *δ* ^18^O values, sample 3 was prepared at *δ* ^18^O_VSMOW‐SLAP_ ~ 45 ‰. These three RMs were carefully measured in multiple batches together with secondary and in‐house water RMs on an LGR Liquid Water Isotope Analyser (described in 2.2.2), and their final isotopic values agreed well within the uncertainty limits with the values calculated based on the gravimetric mixing of USGS46 and IAEA‐607. Table 2 gives an overview for the water RMs that were used in this study.

**TABLE 2:** The *δ* ^18^O (VSMOW‐SLAP) values of selected international water reference materials and prepared water mixtures.

Reference water	*δ* ^18^O_VSMOW‐SLAP_ (‰)
VSMOW2	0.00 ± 0.02^a^
SLAP2	−55.50 ± 0.02^a^
GISP	−24.76 ± 0.09
Sample 1 (close to IAEA‐603)	28.53 ± 0.08^b^
Sample 2 (close to USGS44)	14.67 ± 0.04^b^
Sample 3	44.93 ± 0.04^b^

^a^ The primary RMs VSMOW and SLAP did not have an uncertainty but the recommended secondary RMs for the ^18^O VSMOW‐SLAP scale VSMOW2 and SLAP2 do.

^b^ The uncertainties in *δ* ^18^O_VSMOW‐SLAP_ (‰) in samples 1 to 3 are measurement uncertainties. The water mixtures were measured on three different measurement days and with 50 repetitions (injections) per measurement day.

### 2.2 Instrumentation

#### 2.2.1 Composition of Calcium Carbonate Reference Materials

Qualitative analysis of the composition of calcium carbonate RMs was performed to gain insights in the purity of the RMs. Besides the composition of the RMs, the crystal structure of the calcium carbonate RMs was also explored. A quadrupole time‐of‐flight mass‐spectrometer (QTOF‐MS) was used to investigate if any organic residues were present in the RMs. This was done by mixing small portions of the calcium carbonate RMs in methanol and hexane. The supernatant solutions were then analyzed using a QTOF‐MS. Powder X‐ray diffraction (PXRD) analysis was performed to characterize and establish the predominant crystal structure of CaCO_3_. Element characterization, indicating possible elements that can occur in the form of oxides in the RMs, was conducted by inductively coupled plasma mass spectrometry (ICP‐MS). Significant abundances of elements such as ^24^Mg, ^57^Fe, and ^88^Sr indicate the possibilities of the presence of formed oxides with these elements.

#### 2.2.2 Optical Water Isotope Measurements

The three water mixtures, samples 1 to 3, were analyzed using a Liquid Water Isotope Analyzer (LGR‐LWIA 912‐0050, Los Gatos Research, California, United States, now ABB), to determine the triple‐stable isotope composition: *δ* ^18^O, *δ* ^17^O, and *δ* ^2^H on the VSMOW‐SLAP scale. Sample measurements were bracketed with local references and the international reference waters VSMOW2 and SLAP2.

#### 2.2.3 Weighing

Calcite RMs and additives were weighed on a Mettler Toledo MT5 micro balance, readability 1 μg, weighing capacity 5.1 mg, repeatability 0–2 g: 0.8 μg.

#### 2.2.4 Pyrolysis System and Conditions

Both the calcite RMs and water RMs were completely pyrolyzed using an Elemental Analyzer (EA) (Elementar Vario PYRO Cube CNSOH with the so‐called purge‐and‐trap (described in [25, 26])). All oxygen atoms present within samples were quantitatively converted to carbon monoxide at 1450 °C, and the amount fraction was quantified. The EA is connected in continuous‐flow mode to a stable isotope ratio mass spectrometer (Isoprime PrecisION). The supply and delivery of the monitoring gas are handled by the CentrION system, which is located inside the isotope ratio mass spectrometer. A custom‐built cover was mounted over the sample carousel that introduced a constant flow of N_2_ gas (~100 mL/min.) over all the samples, preventing oxygen and moisture from the surrounding from entering the samples. The EA was operated in oxygen‐only pyrolysis mode. The flow of helium through the pyrolysis reactor was set at 125 mL/min and the temperature of the pyrolysis oven was maintained at 1450 °C. The glassy carbon reactor filling contained reactive carbon in the form of carbon black, glassy carbon, and graphite felt as recommended by the manufacturer. Besides the pyrolysis reactor, the EA also contained a chemical trap for removing water and carbon dioxide (half filled with Sicapent (phosphorus pentoxide with indicator) and sodium hydroxide). Following the replacement of the reactor filling and before samples were pyrolyzed, the system was flushed thoroughly overnight with helium with the reactor set at 1450 °C. This overnight flushing ensured a very low *m/z* 28 background (~1–2 × 10^−11^ nA). Before starting a new measurement sequence, the CO area of blank pyrolysis was always inspected and measurements were only conducted once the CO blanks were below a predefined threshold [27].

The CO formed by the pyrolysis reaction was introduced into the isotope ratio mass spectrometer through the CentrION system and the signal intensities of the ions at *m/z* 28, and *m/z* 30 were measured to obtain a *δ* ^18^O value. To prevent any interference caused by isobaric species [28] or ions that can influence through space‐charge interactions [29], CO was first temporarily retarded in a CO column (a proprietary part from Elementar), while all contaminants were flushed out. Following this, clean helium was introduced through a bypass line into the CO column leading to the TCD (thermal conductivity detector) and later in to the isotope ratio mass spectrometer. The flow of helium through the pyrolysis reactor was diverted and released to the outside to ensure a clean reactor before the next sample was pyrolyzed. Once the contaminants were flushed out, indicated by low and stable baseline currents, CO was released by heating the CO column.

The pyrolysis reactor contained a graphite crucible to collect remains from the additives and the molten silver capsules. After approximately 140 pyrolysis reactions the graphite crucible must be emptied and cleaned, thus keeping the residue built‐up in the graphite crucible low, leading to much better reproducible *δ* ^18^O values from the pyrolysis reaction. In addition, the lifetime of the graphite crucible is longer when cleaned regularly.

### 2.3 Pyrolysis Method for Calcium Carbonate and Water References

The oxygen isotope measurement of calcites by high‐temperature pyrolytic reduction is based on the quantitative conversion of the oxygen from calcite into carbon monoxide. A primary requirement to compare the different materials is to avoid oxygen isotopic fractionation in the pyrolysis process, in other words the thermal decomposition to carbon monoxide of both materials should be 100%. Both materials have challenges to achieve this 100% conversion. For calcite RMs, a complete decomposition is very difficult, despite the high temperature of the pyrolysis oven. The reaction of calcium carbonate to calcium oxide is more favorable than the reaction to carbon monoxide. Silver chloride (AgCl) is a known facilitator for the reaction of calcium carbonate to carbon monoxide [30, 31, 32]. Besides AgCl, which facilitates the reaction of calcium carbonate to carbon monoxide, the presence of an extra carbon source like graphite (C) can increase the yield of carbon monoxide production to 100%. Without adding the additives AgCl and C, the yield will be around 67% only, also noted elsewhere [31, 32]. For this study, AgCl (99.999% trace metals basis, 204382‐5g, Sigma‐Aldrich) and carbon glassy spherical powder (2–12 μm, 99.95%, 484164‐10g, Sigma‐Aldrich) were used as additives, in the ratio of 5:3 (mass ratio). This ratio is similar to that described by Gehre et al. [32] and Boschetti et al. [31].

The calcite RMs were weighed into silver capsules (4 × 3.2 mm, ultra clean) together with the additives. Making a homogeneous mixture of calcite and AgCl and C(s) is challenging and hence, CaCO_3_ and the additive mixture were weighed separately into the same silver capsule. The results with the highest reaction yields were found when the addition was performed in a so‐called sandwich method. First, 1 mg of additive mixture (AgCl/C = 5:3) was put into the capsule, then 0.3 mg calcite was added, followed by 1 mg of additive mixture.

For water RMs, the high temperature of the pyrolysis oven provides enough energy to break the O‐H bond. However, for water RMs, it is key to avoid evaporation losses in the silver capsules that feed the pyrolysis system, as evaporation would lead to isotopic fractionation. Additionally, capsules must be carefully sealed such that air is not co‐trapped within the enclosed capsule.

The “silver capillary tubes technique” developed by the US Geological Survey lab (USGS) solves these issues [33], and thus, the RMs VSMOW2, SLAP2, and GISP in silver tubes were obtained from the USGS lab. In addition, the USGS lab in Reston also kindly prepared silver capillary tubes for us with our water mixtures: samples 1 to 3 (that we supplied to them in quantities of tens of milliliters). Approximately 0.15 μL of water was sealed in each of these silver tubes. The silver water tubes are very small and gave complications in the autosampler as they could slide underneath the sample carousel plate. Therefore, the silver tubes were placed inside a silver capsule and firmly squeezed with a pair of tweezers, to avoid inclusion of air. Obviously, water does not need additives for complete pyrolysis, but to adhere to the rule of identical sample treatment, silver capsules with a silver tube and additives were compared to silver capsules with a silver water tube without additives. No difference in yield or *δ* ^18^O values were observed. Therefore, RM silver tubes inside silver capsules were used without adding additives.

A typical continuous‐flow IRMS mass spectrogram (see Figure S3 in Supporting Information) shows a CO sample peak, followed by two monitoring gas pulses.

To avoid mass dependence issues, so‐called linearity, the amounts of CO from water and calcite RMs in the isotope ratio mass spectrometer have to be as close together as possible. The amount of water in the silver tubes was fixed, so the amount of calcite RM that was weighed into the silver capsules was tuned, such that it would give the same peak areas. Both the formed CO from water and calcite pyrolysis reactions were diluted and only 50% of CO was delivered to the Iisotope ratio mass spectrometerRMS. The typical mass dependence rate of the isotope ratio mass spectrometer for the ratio of ^12^C^18^O to ^12^C^16^O (*m/z* 30 to 28) was between 0.01 and 0.03‰ /nA, and because the signals of our samples varied only between 23 and 26 nA, this influence on the results was negligible.

The pyrolysis yield of the calcite RMs was quantified using silver capsules with only additives (blanks) that were pyrolyzed after a calcite pyrolysis. If a pyrolysis reaction was incomplete, the succeeding blank would show an EA‐TCD signal larger than expected of a blank. And thus, the peak area of the blank was a good indication of the pyrolysis yield of the previous calcite pyrolysis. Therefore, if the blanks with additives after a calcite RM showed no difference compared to blanks with additives in a clean pyrolysis oven (background values), the reaction was considered complete. Another, more coarse check of whether the pyrolysis reaction was complete for calcite RMs was provided by the EA‐derived oxygen amount fraction (*x*(O)) in the materials. Water RMs could not serve as a calibration material for the calculation of the oxygen amount fraction because the amount of water in the silver tubes is only approximately known. For that reason, an easy‐to‐pyrolyze material like benzoic acid (C_6_H_5_COOH, *x*(O) = 26.2%) was added to the pyrolysis series to calibrate the area of the CO peak with the expected *x*(O). Accurate weighing is key for this calculation, so weighing of calcites and benzoic acid was performed on a microbalance (see 2.2.4). If the pyrolysis of the calcite RM is 100%, the measured *x*(O) will be equal to the theoretical value of 48% for calcium carbonate. The uncertainty in the oxygen amount fraction determination using the EA is typically 1–2%, irrespective of the amount fraction of oxygen present in the molecule. This uncertainty limits the use of these oxygen amount fraction for determining the yield of the pyrolysis reaction. However, it is a method that, in addition to the blank method, has increased our confidence that we indeed can achieve a complete pyrolysis of the calcites.

Besides carbon glassy powder as an additional carbon source in the additives mix to facilitate a 100% yield of the calcite RMs, carbon black (also used in the pyrolysis reactor‐filling, product number 200005511, Elementar) and carbon nano powder (<100 nm, 633 100, Sigma‐Aldrich, CAS number 7440‐44‐0), were investigated to ascertain which carbon source would improve the yield the most. Based on the blank method (described above) and the *x*(O) of the calcite reactions, carbon glassy powder (C) was the most effective yield enhancer.

Several tests were performed to find the optimal ratio between calcite and the additives mixture. Data showed that at least a ratio of 1:4 was needed to obtain an oxygen amount fraction near 48% and a low blank in the succeeding sample. Adding the calcite RM between two layers of additives (sandwich method) yielded consistent results, but doubled approximately the total amount of additives and led to very different ratios between calcite and additives compared to the ratios described in Boschetti and Gehre. The total masses used per pyrolysis analysis in this study were 300 μg CaCO_3_/1250 μg AgCl/750 μg carbon glassy powder.

Reaching a 100% conversion from calcium carbonate to carbon monoxide is crucial to minimizing oxygen isotopic fractionation. In the period developing the optimal pyrolysis method, there were many conversions for which the 100% yield was not reached, most noteworthy those where no additives were added to the calcites. As described earlier, we experimented with the ratio between the additives and calcite to increase and optimize the yield, but another important factor for achieving a 100% pyrolysis was the zone in the reactor where the pyrolysis occurred. If the height of the leftover residue of silver and additives was too high in the graphite‐crucible, the sample was dropped in a different temperature zone (lower) and that temperature was not optimal for a complete pyrolysis. We investigated whether the yield to *δ* ^18^O relation followed a Rayleigh isotopic fractionation function [13]. If so, the fractionation factor deduced would give us a good indication of the possible isotopic effect of a yield of not exactly 100%.

To ensure that *δ* ^18^O results of calcite RMs were not affected by a potential contamination by surrounding water, an absorption experiment was conducted. Two sets of dried CaCO_3_ were, prior to pyrolysis analysis, exposed to water with very different *δ* ^18^O values, namely water of *δ* ^18^O = − 55.5‰ and more enriched water of *δ* ^18^O = + 56‰, respectively. Two sets of five samples of CaCO_3_ were weighed out in silver capsules and left open. Each set was placed in a separate metal container. An open Petri dish containing a small layer of water with *δ* ^18^O = −55.5‰ was placed into one of the containers, and a Petri dish containing a similar amount of the more enriched water of *δ* ^18^O = 56‰ was placed into the other. Once the containers were closed, a hairdryer was used to heat the containers to encourage evaporation of the water. These containers were left sealed for ∼24 h. The CaCO_3_ samples were taken out, the foil capsules were properly closed and pyrolyzed. After pyrolyzing, there was no significant difference between the measured *δ* ^18^O values of the two sets. This supports the low hygroscopicity of carbonates [34, 35, 36], and hence, adsorbed water does not pose a risk to our experiments.

One measurement sequence in this study typically consisted of two different calcite RMs, with five replicates each, alternating with blanks with additives. Furthermore, there were three different water RMs with 10 replicates each, and finally a few benzoic acid samples for yield calibration (see Table S2 in the Supporting Information). Experimenting with longer measurement sequences made clear that cleaning the graphite crucible after 140 measurements is crucial for maintaining measurement precision for pyrolysis series which contained calcite RMs and blanks with additives, as those additives were not completely consumed and thus caused more residue in the graphite crucible. This restriction implied that only a selection of the water RMs could be present in one series. Water RMs that were chosen were VSMOW2, sample 1, and sample 2, assuring that *δ* ^18^O values for all the calcites would fall inside this *δ* ^18^O range of water RMs. All water RMs mentioned in Table 2, from SLAP2 (*δ* ^18^O = − 55.5‰) through sample 3 (*δ* ^18^O = 44.93‰) were used to assess the linearity of the *δ* ^18^O_VSMOW‐SLAP_ scale across this broad range. The water scale was linear but with a slope slightly deviating from 1 (0.97) providing an additional reason to limit the *δ* ^18^O_VSMOW‐SLAP_ range from 0 to approximately +30‰. An example of a typical pyrolysis batch sequence is shown in the Supporting Information.

**FIGURE 2:**
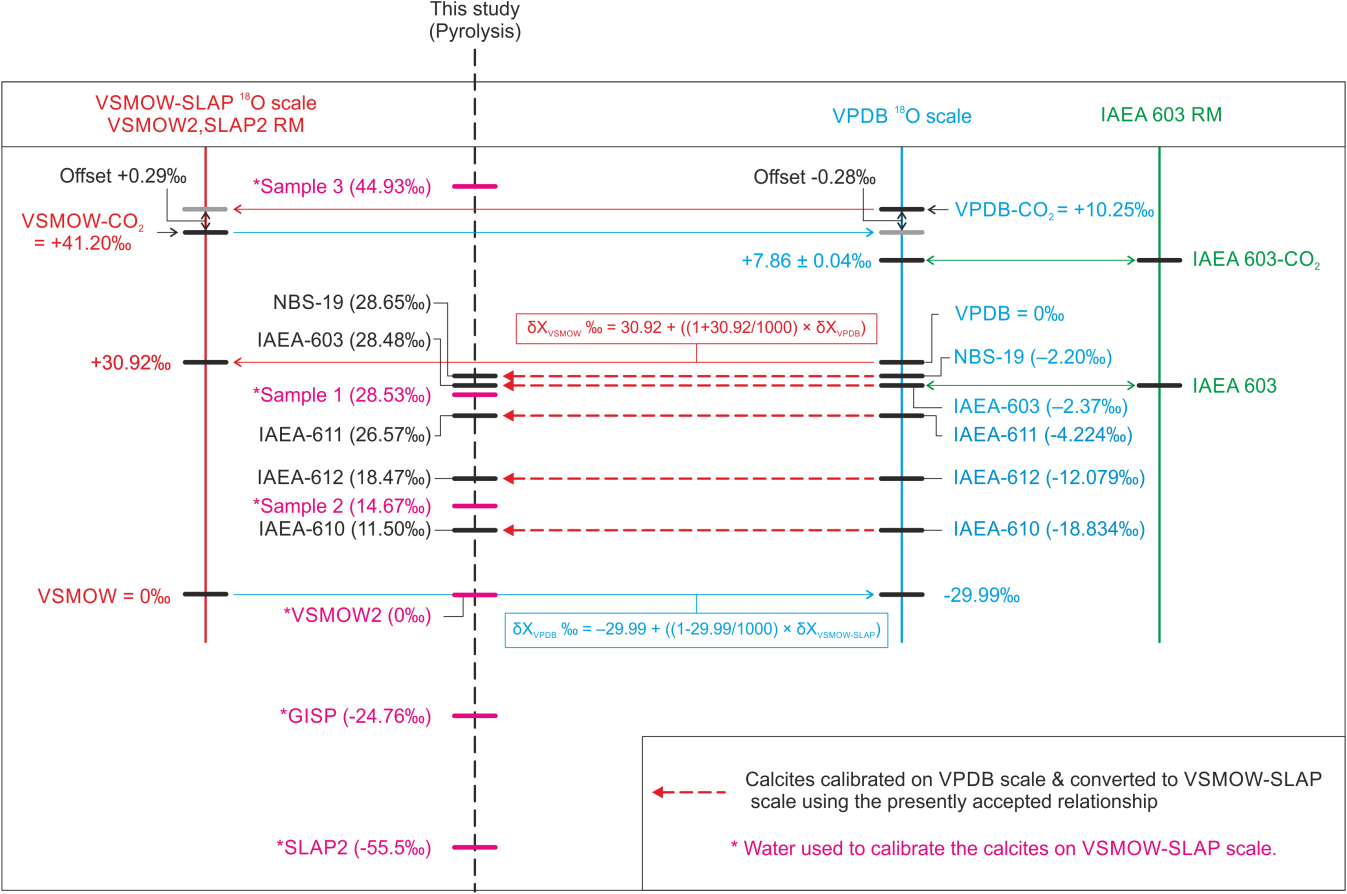
Visualization of the relation between the *δ* ^18^O isotope scales based on water‐CO_2_ equilibration (VSMOW‐SLAP, left, in red) and based on the calcite‐phosphoric acid reaction (VPDB, right, in blue). In blue, the calcium carbonate RMs used in this pyrolysis study are shown with their assigned *δ* ^18^O_VPDB_ values. In pink the water RMs and mixtures that were used in this pyrolysis study are shown on the dotted black vertical *δ* ^18^O_VSMOW‐SLAP_ scale. And on the same scale, in black, the *δ* ^18^O_VSMOW‐SLAP_ values of the calcium carbonate RMs are presented based on the presently accepted relation between the two ^18^O scales, the conversion relation is indicated by red dotted arrows. The water mixtures are indicated on the vertical *δ* ^18^O_VSMOW‐SLAP_ scale to show that they cover the whole range of the carbonate RMs. IAEA‐603 is separately shown in green (most right).

Figure 2 is an extension of Figure 1 and shows the currently recommended relation between the *δ* ^18^O_VPDB_ and *δ* ^18^O_VSMOW‐SLAP_ scale as well as the water references and calcite references used in this pyrolysis study.

### 2.4 Uncertainty Calculation

Many different sources of uncertainty were taken into account in the whole process: The standard deviations of the replicate pyrolysis measurements of the RMs, the uncertainty in the assigned *δ* ^18^O values of the water RMs, the uncertainty of the normalisation fit line through the water RMs, and the uncertainty of the *δ* ^18^O VSMOW‐SLAP scale (due to the uncertainties in VSMOW2 and SLAP2) were all properly taken into account for the calculation of the combined uncertainty for each single experiment. For the final result of the whole experiment, consisting of 50 individual calcite RM measurements, the individual uncertainties of those measurements were uncorrelated (thus of a random nature), and the final uncertainty is thus the standard error of the mean of the averaged results. A detailed description of the uncertainty calculation is available in the Supporting Information.

## 3 Results

### 3.1 Results for the Composition of Calcium Carbonate Reference Materials

Element characterization of the calcium carbonate RMs was carried out by ICP‐MS, resulting in percentages with respect to the total mass for a range of elements. More than 2% of NBS 18 are minerals, with which oxides can be formed, and NBS 18 was for that reason excluded from this study. Table S1 in the Supporting Information shows the ICP results of the calcium carbonate RMs that were tested. Our composition results for NBS 18 and NBS 19 confirmed the previous results from Crowley et al. [37].

The QTOF‐MS technique revealed that the calcium carbonate RMs were not contaminated with any organic residues, which could potentially be a source of oxygen when pyrolyzed together with calcite.

PXRD spectra from the selected RMs, listed in Table 1, were compared with the reference spectra of pure calcite, aragonite, and dolomite. All RMs appeared predominantly, in fact almost exclusively in the calcite phase: their maximum intensity peak was also present in the calcite reference spectrum and characteristic peaks from the dolomite and aragonite reference spectrum were not visible. In Figure S4 of the supporting information, the PXRD spectra are shown together with reference spectra of calcite, aragonite, and dolomite. The confirmation that the RMs were in the calcite form is important, as the isotopic fractionation of the (oversaturated) phosphoric acid reaction shows a dependence on the type of calcium carbonate [19]. As all the tested carbonate RMs were in the calcite phase, PXRD analyses did not lead to any exclusion of specific RMs.

### 3.2 Conversion Yield Pyrolysis

**FIGURE 3:**
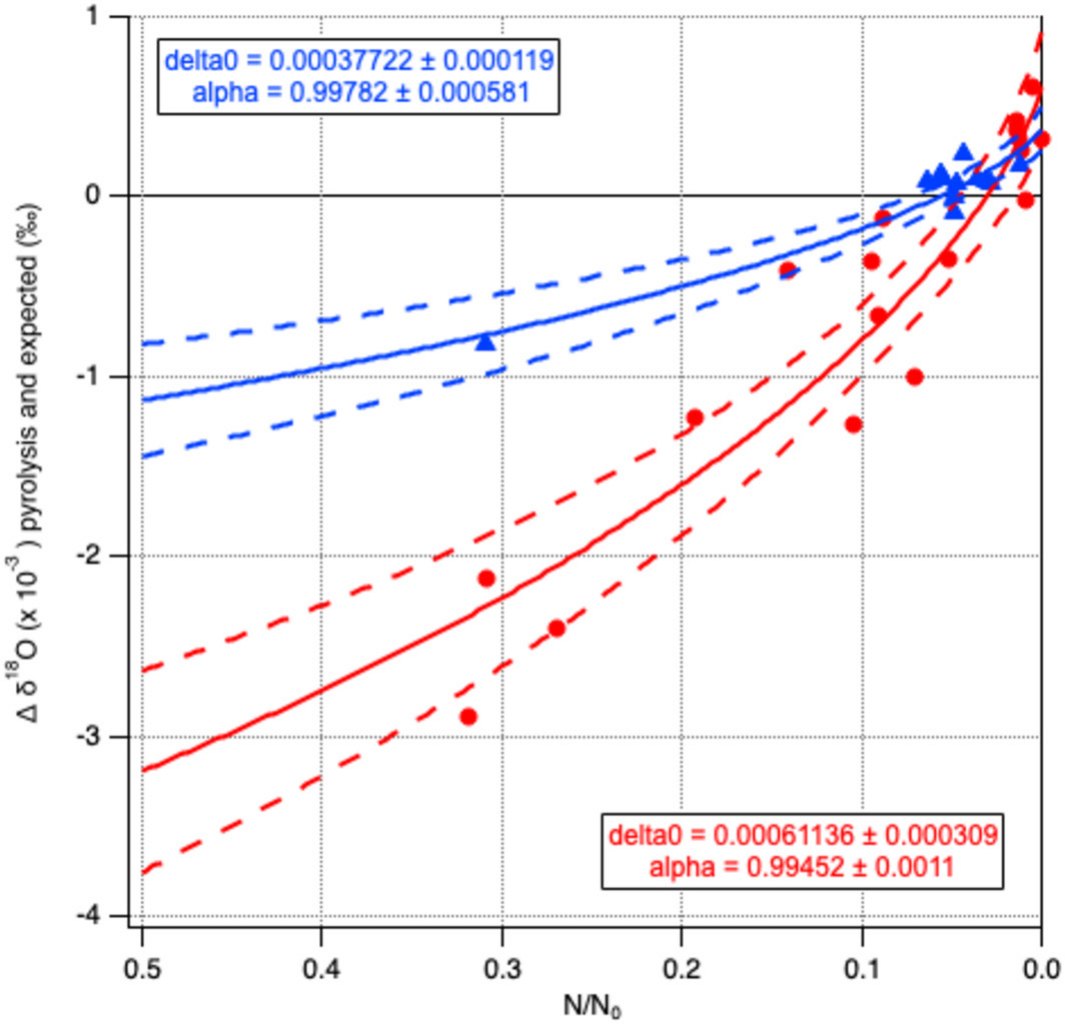
The *δ* ^18^O isotopic Raleigh fractionation against pyrolysis yield. N/N0 represents the yield (*N*/*N*_0_ = 0, when yield is 100%). Data points in blue are from pyrolysis analyses of calcites with the optimized pyrolysis method. Data points in red show larger isotopic fractionation, these calcites pyrolysis measurements are not performed at the optimal temperature or at the optimal ratio between calcites and additives. The datapoints at around *N*/*N*_0_ = 0.3 were from pyrolysis measurements in which no additives were added to the calcite RM (blue and red). The dashed lines show the 95% confidence bands of the fits.

Achieving a 100% conversion yield from the pyrolysis of calcium carbonate to carbon monoxide proved extremely challenging. Consequently, during the pyrolysis optimalization experiments, much of the data was gathered from incomplete pyrolysis reactions, resulting in isotopic fractionation. Figure 3 shows the resulting oxygen isotopic fractionation for all pyrolysis tests, with their varying, incomplete, pyrolysis. In the graph N/*N*_0_ (*x*‐axes) represents the remaining fraction of the original portion of calcite (*N*/*N*_0_ = 0, when yield is 100%). The Rayleigh formula for the fraction removed (the remaining fraction that stayed in the pyrolysis oven), [13], with the fractionation factor α for the conversion reaction as a fit parameter, is used as a fit function for these data (formula is given in the Supporting Information). The figure with the fit shows that the incomplete pyrolysis data points indeed followed, more or less, a Rayleigh curve. At the start of the study, the isotopic fractionation with incomplete conversion yield when varying the ratio of additives to calcite material was larger than when the optimal pyrolysis method was found. The optimal pyrolysis method with the highest conversion yield was achieved by an optimal ratio of additives and calcite material and a completely cleaned out graphite crucible, taking away previous silver‐built‐up more rigorously than before, probably resulting in a more appropriate oven temperature in the sample‐drop‐zone. A well‐known phenomenon is that isotopic fractionation is smaller with higher temperatures. This smaller isotopic fractionation effect will make the method much more robust, even if the conversion yield is not exactly 100%. The datapoints with the optimized method are shown in blue, with one datapoint at around *N*/*N*_0_ = 0.3 when no additives were added. The data points found in the period of experimentation with the additives (and initially less care was taken to clean the graphite crucible thoroughly) are shown in red, datapoints around *N*/*N*_0_ = 0.3 again without additives. From the graph it can be clearly seen that the fractionation factor alpha is closer to 1 for the optimized method. The Rayleigh function fit values are indicative only, as the calculation of the yield is based on the measured oxygen yields of the pyrolysis reactions. As mentioned in Section 2.3, these oxygen yields are not precise leading to larger uncertainties, but the data showed a consistent pattern of isotopic fractionation when the conversion yield is not complete.

The Rayleigh fit provides the fractionation factor α for the pyrolysis reaction, 0.9978 ± 0.0006 (*k* = 2), or the fractionation ε being −2.2 ± 0.6‰. This fractionation implies that, if we strive for a maximum deviation of 0.2‰ in *δ* ^18^O, the reaction should have a yield better than 97.6%. Based on our pyrolysis results, specifically the negligible yields of the blanks with additives alternating with the calcite samples, we are confident that the yield we achieve is higher than this value.

**FIGURE 4:**
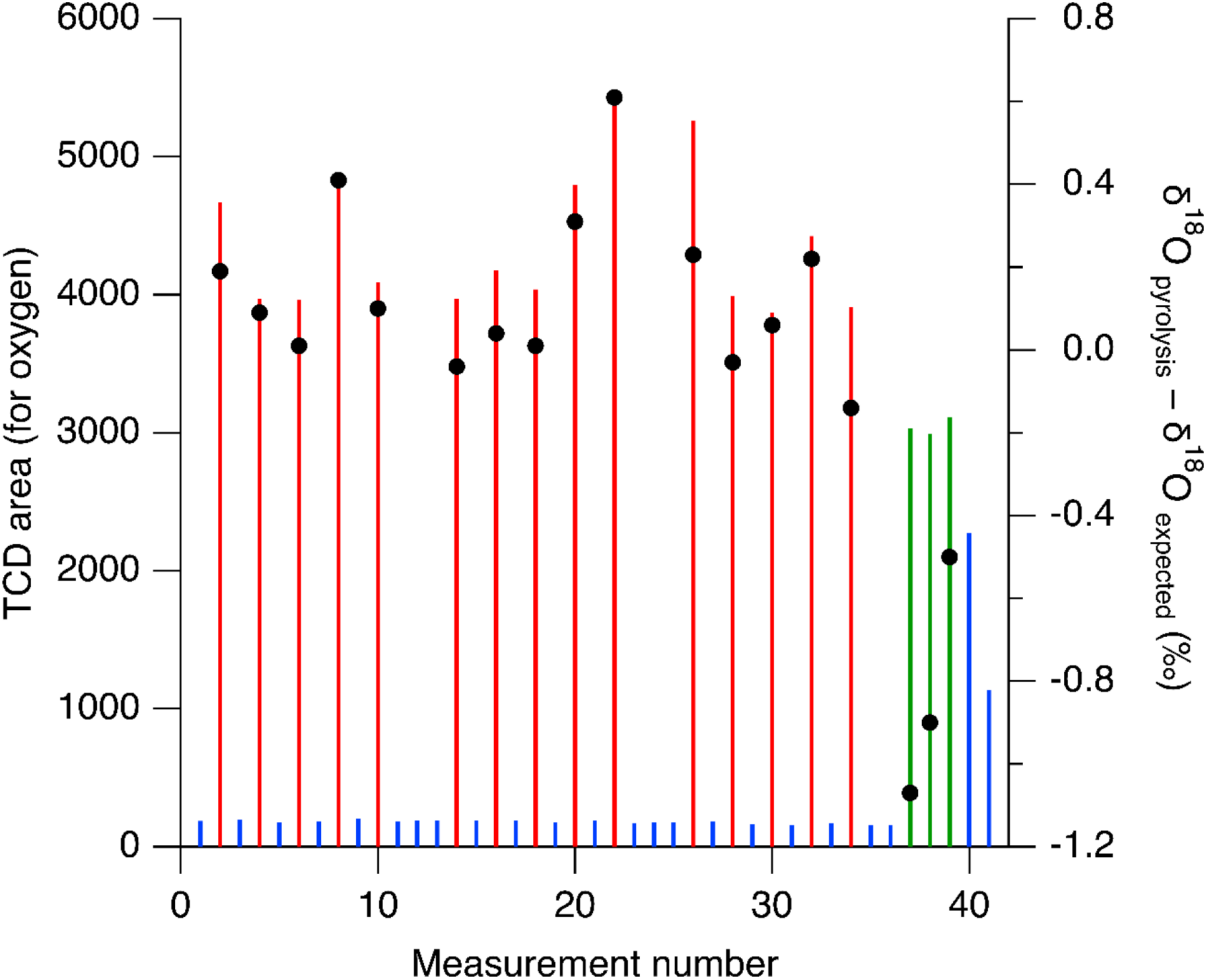
The oxygen area in TCD units from three different calcite RMs with additives (red) (*n* = 5) and succeeding blanks with additives (blue) (left *y*‐axes) and *δ* ^18^O difference from the pyrolysis and the expected *δ* ^18^O values for those calcite RMs (right *y*‐axes, black dots). The last three calcite RMs were pyrolyzed without additives (green, measurement number 37–39), resulting in far from complete pyrolysis, visible in the large succeeding blanks and the isotopic fractionation.

In a specific measurement sequence, three water references (*n* = 10) were pyrolyzed together with two different calcite RMs (with additives added) (*n* = 5). Alternating with the individual calcite RMs pyrolysis, blanks with additives were pyrolyzed. The pyrolysis reaction was considered to be 100% when the succeeding blank after the calcium carbonate pyrolysis had the same O peak area as a blank in a clean pyrolysis oven. An accidental higher oxygen TCD area of a succeeding blank with approximately 5% of the previous calcite RM, showed no measurable isotopic fractionation effect, supporting the robustness of the method, and indicating that the requirement of > 97.6% based on the Rayleigh fractionation fit is too strict. In Figure 4, the oxygen areas are shown in red (EA TCD) from three different calcite RMs with additives (five replicate measurements), each calcite RM was succeeded by a blank with additives (shown in blue). All the blanks with additives, succeeding calcite RM with additives had a low TCD area, indistinguishable from the area of the blanks with additives in a clean oven, supporting a 100% yield of the previous calcite pyrolysis measurements. In contrast, at the end of the series one calcite RM without additives (shown in green) was performed in triplicate, succeeded by two blanks with additives. As can be seen clearly, the last two blanks show very high oxygen areas as the conversion yield from the previous calcite RM without additives was only approximately 67%. On the right *y*‐axes of Figure 4, the difference in *δ* ^18^O values from pyrolysis and from what was expected based on the literature relation between VPDB and VSMOW‐SLAP scale is shown. In the cases with the pyrolysis calcite RM measurements without additives, where the pyrolysis yield was about 67%, a significant isotopic effect is visible, resulting in a much lower *δ* ^18^O value, confirming the outcome shown in Figure 3. In Figure 4, the results for the RMs with additives average to a *δ* ^18^O_pyrolysis‐expected_ value of 0.14 ± 0.19‰ (standard deviation).

### 3.3 Analysis Pyrolysis Measurement Sequence

**FIGURE 5:**
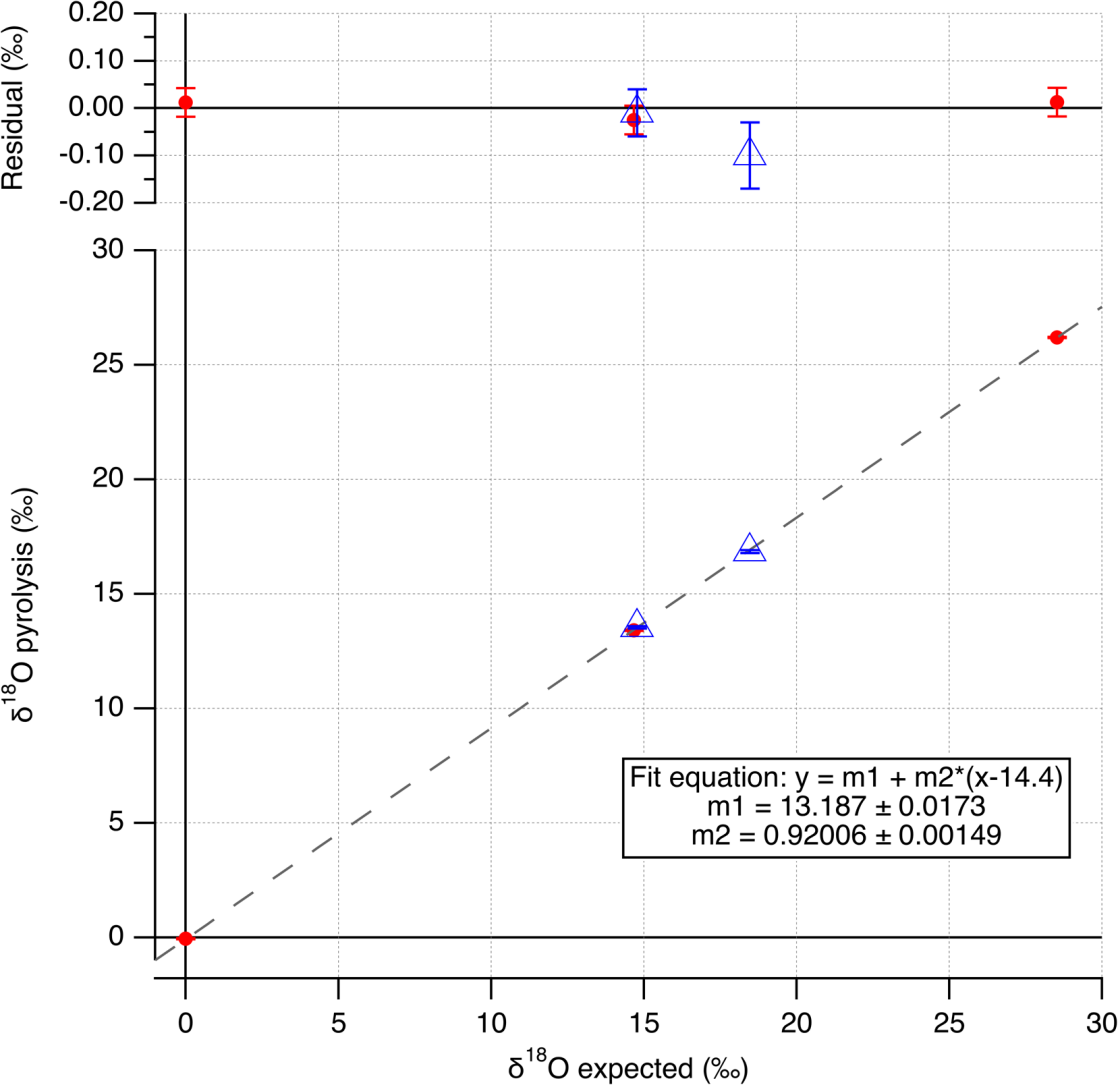
Normalization fit line of the water references (red) and the calculated *δ* ^18^O of the calcite RMs (blue) (*n* = 5 per calcite RM). On top, the residuals are shown.

For analyzing a full pyrolysis measurement sequence (“batch”), the measured *δ* ^18^O_VSMOW‐SLAP_ of the water RMs was plotted against the assigned *δ* ^18^O_VSMOW‐SLAP_. The *δ* ^18^O_VSMOW‐SLAP_ of the calcite in the same pyrolysis batch can be calculated by using the three‐point linear‐normalization fit line based on the relation between the measured and the assigned *δ* ^18^O_VSMOW‐SLAP_ values of the water references. In Figure 5, this fitting process is visualized for one specific pyrolysis batch, and in Table 3, the data for this pyrolysis batch are shown. To ensure that the uncertainties in the slope and the intercept of the normalization fit line are independent of each other, the intercept point of the fit is put at the point of gravity of the x‐axes, *δ* ^18^O_expected_, in this case 14.4‰.

**TABLE 3:** The *δ* ^18^O_VSMOW‐SLAP_ pyrolysis measurements for one specific measurement sequence with three water RMs and two calcite RMs are shown. The assigned *δ* ^18^O_VSMOW‐SLAP_ values are the published value for VSMOW2 and the listed values in Table 2 for the water mixtures. The literature *δ* ^18^O_VSMOW‐SLAP_ values for the calcite RMs are based on the assigned (published) *δ* ^18^O_VPDB_ values and the current conversion relation between *δ* ^18^O_VPDB_ and *δ* ^18^O_VSMOW‐SLAP_. The *δ* ^18^O_VSMOW‐SLAP_ fitted values are based on the normalization fit line through the water RMs. Between brackets the standard deviation (SD) or the standard error in the mean (SEM) is shown. For the calcites the *δ* ^18^O_VSMOW‐SLAP_ values were normalized based on the fitline through the water RMs. The combined uncertainty includes the uncertainty of the normalization fit line and the SD of the five replicate measurements.

Name	*δ* ^18^O_VSMOW‐SLAP raw_ (SD, 1σ) pyrolysis	n	Assigned *δ* ^18^O_VSMOW‐SLAP_ (SEM)	Literature *δ* ^18^O_VSMOW‐SLAP_ (SEM)	*δ* ^18^O_VSMOW‐SLAP_ fitted (SEM)	*δ* ^18^O_VSMOW‐SLAP_ calcites (combined uncertainty)
VSMOW2	−0.05 (0.12)	10	0.00 (0.02)		0.01 (0.03)	
Sample 1	26.20 (0.06)	10	28.53 (0.08)		28.53 (0.03)	
Sample 2	13.41 (0.07)	10	14.67 (0.04)		14.64 (0.03)	
IAEA‐612	16.85 (0.13)	5		18.47 (0.06)		18.37 (0.13)
USGS44	13.54 (0.07)	5		14.78 (0.02)		14.77 (0.08)

### 3.4 Pyrolysis Results

In total, five successful pyrolysis sequences were performed with water and calcite RMs. In Table 4, the *δ* ^18^O_VSMOW‐SLAP_ results for six different CaCO_3_ RMs are shown, as well as the number of independent measurements of one CaCO_3_ RM (each of them consisting of five individual measurements). In case there were multiple measurements, the measurement outcome is averaged (weighted on the calculated combined uncertainty).

**TABLE 4:** Difference in *δ* ^18^O_Pyrolysis_ and *δ* ^18^O_Expected_ for selected calcite RMs.

Reference material	Unit id.	*δ* ^18^O_VSMOW‐SLAP_ (‰) expected	Averaged difference pyrolysis minus expected *δ* ^18^O_VSMOW‐SLAP_ (‰) (SEM)	Number of replicates
IAEA‐603	1007	28.48	0.072 (0.035)	3 × 5
NBS 19	—	28.65	0.17 (0.19)	1 × 5
IAEA‐610	102	11.50	0.111 (0.024)	2 × 5
IAEA‐611	188	26.57	0.09 (0.13)	1 × 5
IAEA‐612	135	18.47	0.025 (0.078)	2 × 5
USGS44^a^	—	14.78 14.68	−0.009 (0.035) 0.091 (0.035)	1 × 5

^a^ As previously stated in Table 1, BGC‐lab from Jena and CIO from Groningen found different *δ* ^18^O values for USGS44 [22], CIO *δ* ^18^O_VSMOW‐SLAP_ value = 14.78‰ (*δ* ^18^O_VPDB_ = − 15.66‰), BGC *δ* ^18^O_VSMOW‐SLAP_ value = 14.68‰ (*δ* ^18^O_VPDB_ = −15.75‰).

**FIGURE 6:**
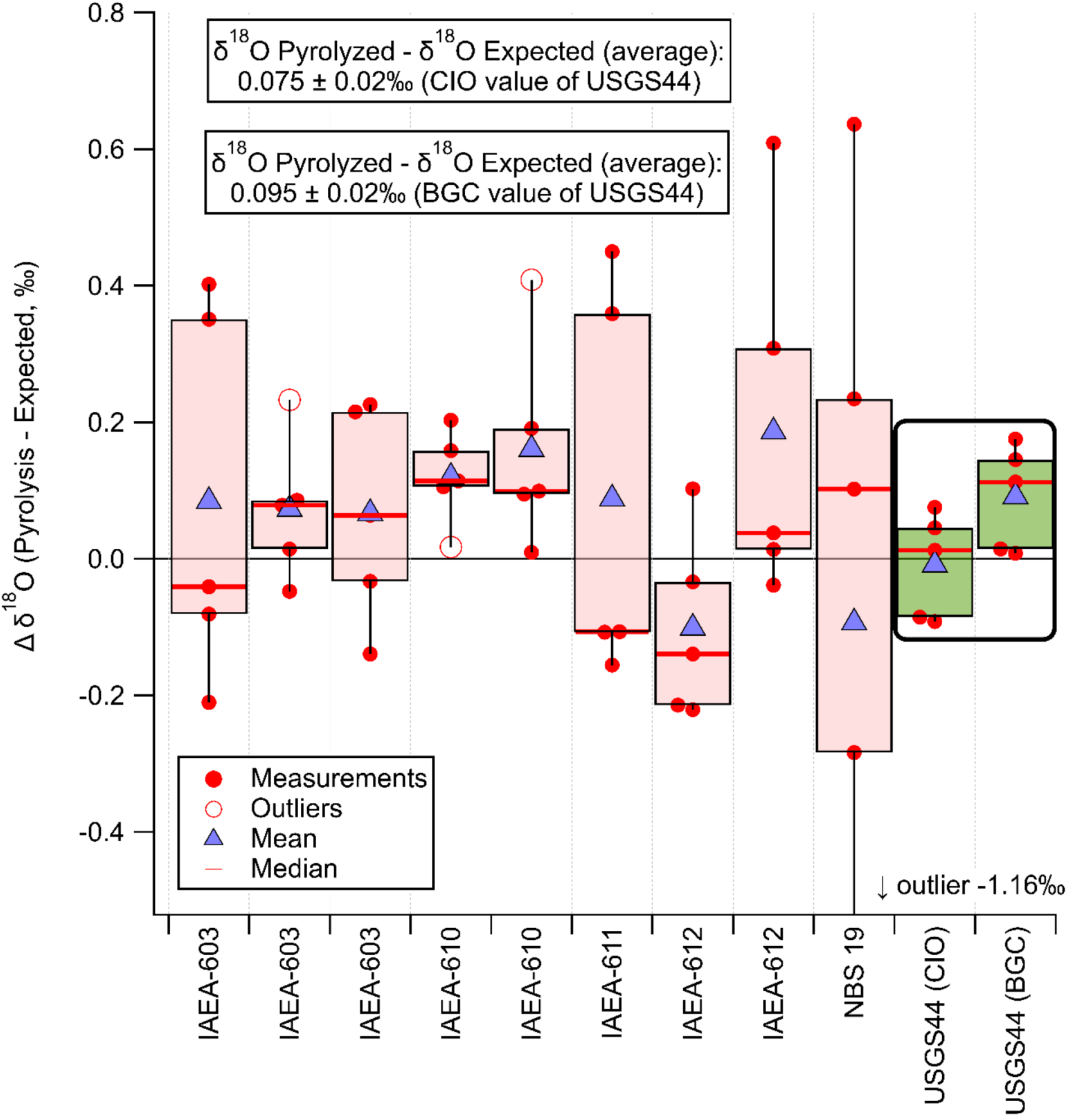
Boxplot of difference in *δ* ^18^O_Pyrolysis_ and *δ* ^18^O_Expected_ values (based on the currently recommended relation between the *δ* ^18^O_VSMOW‐SLAP_ and *δ* ^18^O_VPDB_ scales) for the calcite RMs under study (*n* = 5 per calcite RM measurement). The weighted average (the weight is the combined uncertainty of the SEM of the 5 measurements and the uncertainty resulting from the normalization on the water references) for the difference (Δ) in *δ* ^18^O_pyrolysis_ and *δ* ^18^O_Expected_ values of 47 measurements (50 measurements with 3 outliers, also the outlier for IAEA‐603 is added, because this data point fits well in the complete data set for IAEA‐603) is 0.075‰ ± 0.021‰ when the CIO value for USGS44 is used and 0.095‰ ± 0.019‰ when the BGC value for USGS44 is used.

Figure 6 shows a boxplot of all calcite RMs measurements ordered per reference material. In total, we performed 50 individual calcite reference material measurements. In Figure 6, the outliers are visible as well. Three data points are considered as outliers, shown in Figure 6, since they fall outside 1.5 times the interquartile range (IQR, range between 25th and 75th percentile). One data point for IAEA‐603 fitted well in the complete data set for IAEA‐603 and was not considered as outlier.

Upon considering all CaCO_3_ RM measurements (Table 4 and Figure 6), the average difference of the measured *δ* ^18^O_VSMOW‐SLAP_ values from pyrolysis and the *δ* ^18^O_VSMOW‐SLAP_ values that we expect, based on the currently described conversion relation between *δ* ^18^O_VSMOW‐SLAP_ and *δ* ^18^O_VPDB_ scale, which is 0.075 ± 0.021‰ (USGS44 CIO *δ* ^18^O_VSMOW_ value), or 0.095 ± 0.019‰ (USGS44 BGC *δ* ^18^O_VSMOW_ value). Considering only the recommended RM to realize the *δ* ^18^O calcite scale with the lowest uncertainty, IAEA‐603, 0.072 ± 0.035‰ (*n* = 15) is found for this conversion difference. Based on IAEA‐603 only, a slight offset from the conventional conversion relation is on the brink of 95% significance. In addition, the other CaCO_3_ RMs support, and enhance the precision of, a slight offset in the conversion relation.

The averaged result for the first IAEA‐612 (*n* = 5) deviates from all the other values (see Figure 6), but there was no reason to mark these measurements as outliers.

Unfortunately, the uncertainty in the measurement for NBS 19, the material originally defining the VPDB scale, is quite large, caused by a considerable spread in the five pyrolysis measurements. As the averaged result is weighted with the combined uncertainty, NBS 19 hardly contributed to the final result. Within their uncertainty, however, the NBS 19 results agree with the above values.

## 4 Discussion and Recommendations

**FIGURE 7:**
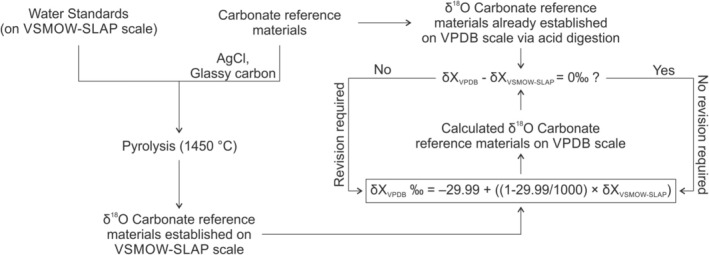
Summarizing flow scheme of steps and decisions performed in this study.

Oxygen isotope measurements are performed on different isotopic scales and the connection between the different scales is not optimally known. As noted in the introduction, currently no direct link between the *δ* ^18^O_VSMOW‐SLAP_ and the *δ* ^18^O_VPDB_ scale is provided in literature. The presently recommended conversion equations from one ^18^O scale to the other [5] are through CO_2_ released (using specified procedures) from different sample materials. This study provides a direct link between the *δ* ^18^O_VSMOW‐SLAP_ and the *δ* ^18^O_VPDB_ scale, and we find a small discrepancy between our results and the currently recommended relation. In Figure 7, the steps and decisions that were made in this study are shown.

In Table 5, the averaged difference (Δ) of *δ* ^18^O_Pyrolysis_ and *δ* ^18^O_Expected_ values (literature) is shown, when different calcite RMs are taken into account. When using the *δ* ^18^O measurements for IAEA‐603 only, (also the outlier from boxplot Figure 6 is added, because this data point fits well in the complete data set for IAEA‐603) the so‐called “zero‐points” will change by +0.07‰. This would lead to the following changed conversion equations between the two ^18^O scales, (all values in ‰) for a sample X:

δO18X,VSMOW−SLAP=30.99+1+30.991000*δO18X,VPDB‰

δO18X,VPDB=−30.06+1+−30.061000*δO18X,VSMOW−SLAP‰

**TABLE 5:** Differences in *δ* ^18^O_Pyrolysis_ and *δ* ^18^O_Expected_ for multiple combinations of calcite RMs in this study, leading to different so‐called “zero‐points.” The parameters in conversion relation between two ^18^O scales are shown.

	Δ *δ* ^18^O_Pyrolysis‐Expected_ (SEM)	Zero‐point VPDB➔VSMOW‐SLAP	Zero‐point VSMOW‐SLAP➔VPDB
Currently recommended conversion between ^18^O scales		30.92	−29.99
IAEA‐603 only (n = 15)	0.072 (0.035)	30.99	−30.06
All calcite RMs (BGC value for USGS44)	0.095 (0.019)	31.02	−30.09
All calcite RMs (CIO value for USGS44)	0.075 (0.021)	31.00	−30.07
All calcite RMs except USGS44	0.096 (0.022)	31.02	−30.09
IAEA‐612 first 5 measurements considered as outliers (BGC value for USGS44)	0.110 (0.018)	31.03	−30.10
IAEA‐612612 first 5 measurements considered as outliers (CIO value for USGS44)	0.089 (0.020)	31.01	−30.08

Considering all calcite RMs that were investigated in this study, with or without USGS44 results, leads to slightly different values for the so‐called “zero‐points”. Table 5 shows all combinations.

The small positive discrepancy from zero in the difference between the *δ* ^18^O measured through pyrolysis and expected values might lead to an adaption of the conversion equations from *δ* ^18^O_VPDB_ to *δ* ^18^O_VMOW‐SLAP_ and vice versa. Then, however, the other values: the offsets and/or the fractionation factors, in the diagram (Figure 1) would need adjustment and should be reconsidered. The uncertainty in the offsets is not given in Hillaire et al. [5], but our laboratory [17] reported 0.32‰ in the past instead of 0.28–0.29‰. A NIST cross‐contamination ring test [38] resulted in *δ* ^18^O differences between both scales, of 0.17 ± 0.18‰, showing the large spread between the seven expert participants (four results lie more closely around −0.25‰). If we assume the fractionation factors to be correct, our results will lead to an offset between the two CO_2_’s of 0.36‰ instead of the 0.29‰ given in Hillaire‐Marcel et al. [5]. As this is the only value that can be measured directly by a laboratory that can reproduce both scales (such as our own lab), such a new measurement, along with proper uncertainty considerations is recommended. On the other hand, the fractionation factors from water to CO_2_ and from calcite to CO_2_ were investigated decades ago (e.g., before general awareness of scale contraction effects), and might have considerably more bias than recognized.

Despite the fact that determination of fractionation factors was based on historical *δ* ^18^O measurements, based on a very different and complicated experiment (quantitatively converting water into oxygen gas (by fluorination) and then into carbon dioxide), and scale‐contraction was not fully recognized at the time of these experiments, still, the outcome from this study showed a small discrepancy only, which is quite reassuring.

Performing experiments on only one ^18^O scale avoids the necessity of comparing results to another ^18^O scale, and therefore, this study will not induce in‐scale modifications. But as the *δ* ^18^O_VPDB_ scale is an artifact‐based scale, anchoring this *δ* ^18^O_VPDB_ to the *δ* ^18^O_VMOW‐SLAP_ scale provides a stronger base for *δ* ^18^O_VPDB_ scale. The absolute ^18^O abundance of VSMOW has been determined by Baertschi [20], and the quantitative difference in *δ* ^18^O between VSMOW and SLAP by Aerts‐Bijma et al. [21]. These two efforts make the *δ* ^18^O_VSMOW‐SLAP_ scale, in principle, S.I.‐traceable. However, Baertschi's work is almost 50 years old and using their value by Aerts‐Bijma et al. [21] led to a surprisingly negative value for SLAP. Therefore, it might be worthwhile to repeat the determination of the absolute ^18^O abundance of VSMOW with currently available newer and more precise instruments. As water isotopes can now be easily measured optically (thus also avoiding the H_2_O‐CO_2_ fractionation process), precise and accurate spectroscopic measurements might lead to a new absolute measurement of the ^18^O abundance, in a fashion similar to the work of Fleisher et al. [39] for ^13^C.

According to Assonov [40], some of the calcite RMs tested in this study, namely IAEA‐610, IAEA‐611, and IAEA‐612, are not suitable as *δ* ^18^O reference materials, because they are fine‐powdered and their *δ* ^18^O values might suffer from a vigorously, acid digestion reaction with difficulties of maintaining the temperature constant, or they might suffer from absorption of local moisture both causing *δ* ^18^O alteration. Moreover, *δ* ^18^O_VPDB_ values that were given in the paper were not scaled to the *δ* ^18^O_VSMOW‐SLAP_ scale and the authors mentioned, *δ* ^18^O values were given for information purpose only. Nevertheless, the results that we found in this study corresponded well with these informative numbers, meaning that the purpose of these three reference materials might be reconsidered. In our case, the acid digestion reaction of course did not play a role.

Unfortunately, NBS 19, the primary RM for the *δ* ^18^O_VPDB_ scale, showed a large spread in the measured *δ* ^18^O_VSMOW‐SLAP_ values. Inhomogeneity of the material is not likely to be the cause, as according to Ishimura et al. [10], NBS 19 is homogeneous at grain‐size level. Within the uncertainty, however, the NBS 19 results agree with the found outcome from this study.

In this paper, we consistently use, and refer to, the water ^18^O scale as the VSMOW‐SLAP ^18^O scale [4]. In the recommendations of Hillaire et al. [5] is mentioned that the VSMOW ^18^O scale is defined by two secondary reference materials, VSMOW2 and SLAP2 such that *δ* ^18^O_VSMOW2_ = 0.00 ± 0.02‰ and *δ* ^18^O_SLAP2_ = −55.50 ± 0.02‰. We prefer to consistently mention the *δ* ^18^O water scale the *δ* ^18^O_VSMOW‐SLAP_ scale, to make clear that we applied a two‐point calibration. Being clear about using a two‐point scale is important, as Aerts‐Bijma et al. [21] reported a much more negative *δ* ^18^O for SLAP2, implying that the official VSMOW‐SLAP scale is contracted, and that 1‰ real difference in *δ* ^18^O values would be reported as a 0.985‰ (56.33/55.5) difference.

## 5 Conclusions

This study shows the direct comparison of the two different ^18^O scales for calcium carbonate and water samples through pyrolysis. Our results suggest a small adjustment of the presently recommended relation between the VPDB^18^O scale and the VSMOW‐SLAP ^18^O scale is needed. The results of the secondary defining reference material IAEA‐603 of the VPDB^18^O scale showed a positive deviation of 0.072 ± 0.035‰ from the currently described relationship between the VPDB and VSMOW‐SLAP ^18^O scales. The results of the other calcite RMs in this study namely IAEA‐610, IAEA‐611, IAEA‐612, and USGS44 confirmed this positive adjustment and contributed to a more precise outcome of 0.075 ± 0.021‰. By direct anchoring the two ^18^O scales through pyrolysis and by avoiding a conversion of the reference material to CO_2_ first, the whole scheme of conversion equations from one ^18^O scale to the other is more robust. The findings reported here might lead to a small adjustment of the commonly accepted conversion values of the so‐called zero‐points from one ^18^O scale to the other.

## Author Contributions

**Anita Th. Aerts‐Bijma:** conceptualization; methodology; investigation; data curation; formal analysis; visualization; writing – original draft; project administration; validation; resources. **Dipayan Paul:** conceptualization; methodology; investigation; supervision; visualization; writing – review and editing; validation; resources. **Albert C. van Buuren:** investigation; methodology. **Vivian R Kroon:** investigation; methodology. **Harro AJ Meijer:** conceptualization; methodology; supervision; writing – review and editing; validation; resources.

## Supporting information


**Data S1** Supporting information.

**Table S1** ICP‐MS results for 9 different elements in mass percentages for the calcite RMs. Based on these results, NBS‐18 was not used in this study.

**Table S2** Typical measurement sequence pyrolysis.

**Figure S3** The upper part of the figure is a typical isotope ratio mass spectrometer mass spectrogram of CO, in red m/z 28 and in blue m/z 30. The bottom part of the figure is an EA TCD spectrum with the CO peak while the CO column was heated. The two spikes in the TCD spectrum are caused by switching valves.

**Figure S4** PXRD spectra of all the calcium carbonate references used in this study shown together with the reference spectra of calcite, dolomite, and aragonite. For the purpose of visual clarity, a *y*‐axis offset has been added. As is evident from the data, all materials are virtually pure calcite. The aragonite structure is fully absent, and so is dolomite, except perhaps for a small fraction in NBS 18 (which material has not been used in this study).

## Data Availability

The data presented in this study are openly available through the online repository DataVerseNL at https://doi.org/10.34894/3NMVRH.

## References

[1] G Hut , “Consultant Group Meeting on Stable Isotope Reference Samples for Geochemical and Hydrological Investigations,” IAEA, Vienna 16‐18 September 1985, April (1987).

[2] R. Gonfiantini , “Standards for Stable Isotope Measurements in Natural Compounds,” Nature 271, no. 5645 (1978): 534–536, 10.1038/271534a0.

[3] IAEA Reference Sheet for International Measurement Standards , “VSMOW2 Vienna Standard Mean Ocean Water 2, Water (δ2HVSMOW‐SLAP, δ18OVSMOW‐SLAP) SLAP2 Standard Light Antarctic Precipitation 2, Water (δ2HVSMOW‐SLAP, δ18OVSMOW‐SLAP),” RS_VSMOW2_SLAP2_rev1/2017‐07‐11:1–8

[4] F. Camin , D. Besic , P. J. Brewer , et al., “Stable Isotope Reference Materials and Scale Definitions—Outcomes of the 2024 IAEA Experts Meeting,” Rapid Communications in Mass Spectrometry 39, no. 14 (2025): e10018, 10.1002/rcm.10018. 40275550 PMC12022467

[5] C. Hillaire‐Marcel , S. T. Kim , A. Landais , et al., “A Stable Isotope Toolbox for Water and Inorganic Carbon Cycle Studies,” Nature Reviews Earth and Environment 2, no. 10 (2021): 699–719, 10.1038/s43017-021-00209-0.

[6] W. A. Brand , T. B. Coplen , J. Vogl , M. Rosner , and T. Prohaska , “Assessment of International Reference Materials for Isotope‐Ratio Analysis (IUPAC Technical Report),” Pure and Applied Chemistry 86, no. 3 (2014): 425–467, 10.1515/pac-2013-1023.

[7] S. Assonov , M. Groening , A. Fajgelj , J. F. Hélie , and M. C. Hillaire‐ , “Preparation and Characterisation of IAEA‐603, a New Primary Reference Material Aimed at the VPDB Scale Realisation for *δ*13C and *δ*18O Determination,” Rapid Communications in Mass Spectrometry 34 (2020): e8867, 10.1002/rcm.8867. 32567208

[8] K. Nishida and T. Ishimura , “Grain‐Scale Stable Carbon and Oxygen Isotopic Variations of the International Reference Calcite, IAEA‐603,” Rapid Communications in Mass Spectrometry 31, no. 22 (2017): 1875–1880, 10.1002/rcm.7966. 28833709

[9] I. Friedman , J. O'neil , and G. Cebula , “Two new Carbonate Stable Isotope Standards,” Geostandards Newsletter 6 (1982): 11–12, 10.1111/j.1751-908X.1982.tb00340.x.

[10] T. Ishimura , U. Tsunogai , and F. Nakagawa , “Grain‐Scale Heterogeneities in the Stable Carbon and Oxygen Isotopic Compositions of the International Standard Calcite Materials (NBS 19, NBS 18, IAEA‐CO‐1, and IAEA‐CO‐8),” Rapid Communications in Mass Spectrometry 22, no. 12 (2008): 1925–1932, 10.1002/rcm.3571. 18484681

[11] J. M. McCrea , “On the Isotopic Chemistry of Carbonates and a Paleotemperature Scale,” Journal of Chemical Physics 18, no. 6 (1950): 849–857, 10.1063/1.1747785.

[12] S. Epstein and T. Mayeda , “Variation of O^18^ Content of Water From Natural Sources,” Geochimica et Cosmochimica Acta 4, no. 5 (1953): 213–224, 10.1016/0016-7037(53)90051-9.

[13] WG Mook , “Environmental Isotopes in the Hydrological Cycle: Principles and Applications,” Volume 1, Introduction‐theory, methods, review. *IAEA*. (2000).

[14] I. Friedman and J. R. O'Neil , “Compilation of Stable Isotope Fractionation Factors of Geochemical Interest,” in Data of Geochemistry, vol. 6. Chapter KK, ed. M. Fleischer (U.S. Government Printing Office, 1977).

[15] J. R. O'Neil , L. H. Adami , S. Epstein , and M. Park , “Revised Value for the ^18^O Fractionation Between CO_2_ and H_2_O at 25°C,” Journal of Research of the U.S. Geological Survey 3, no. 4 (1975): 623–624.

[16] T. B. Coplen , C. Kendall , and J. Hopple , “Comparison of Stable Isotope Reference Samples,” Nature 302 (1983): 236–238.

[17] H. A. J. Meijer , “Stable Isotope Quality Assurance Using the ‘Calibrated IRMS' Strategy,” Isotopes in Environmental and Health Studies 45, no. 2 (2009): 105–163, 10.1080/10256010902869113. 20183228

[18] TB Coplen , JA Hopple , JK Böhlke , et al., “Compilation of Minimum and Maximum Isotope Ratios of Selected Elements in Naturally Occurring Terrestrial Materials and Reagents,” (2002), Page 36, Last accessed March 2025, https://pubs.water.usgs.gov/wri014222.

[19] S. T. Kim , T. B. Coplen , and J. Horita , “Normalization of Stable Isotope Data for Carbonate Minerals: Implementation of IUPAC Guidelines,” Geochimica et Cosmochimica Acta 158 (2015): 276–289, 10.1016/j.gca.2015.02.011.

[20] P. Baertschi , “Absolute ^18^O Content of Standard Mean Ocean Water,” Earth and Planetary Science Letters 31, no. 3 (1976): 341–344, 10.1016/0012-821X(76)90115-1.

[21] A. T. Aerts‐Bijma , A. C. van Buuren , D. Paul , and H. A. J. Meijer , “The Absolute *δ* ^18^O Value for SLAP With Respect to VSMOW Reveals a Much Lower Value Than Previously Established,” Rapid Communications in Mass Spectrometry 38, no. 6 (2024): e9678, 10.1002/rcm.9678. 38356090

[22] H. Qi , H. Moossen , H. A. J. Meijer , et al., “USGS44, a New High‐Purity Calcium Carbonate Reference Material for *δ*13C Measurements,” Rapid Communications in Mass Spectrometry 35, no. 4 (2021): e9006, 10.1002/rcm.9006. 33201519 PMC7816275

[23] V. Faghihi , B. M. A. A. Verstappen‐Dumoulin , H. G. Jansen , et al., “A New High‐Quality Set of Singly (^2^H) and Doubly (^2^H and ^18^O) Stable Isotope Labeled Reference Waters for Biomedical and Other Isotope‐Labeled Research,” Rapid Communications in Mass Spectrometry 29 (2015): 311–321, 10.1002/rcm.7108. 26406342 PMC6680334

[24] TB Coplen , “Report of Stable Isotopic Composition. Reference Material USGS46 Ice Core Water (Hydrogen and Oxygen Isotopes in Water),” (2019), last accessed Oktober 2024, https://www.usgs.gov/media/files/rsil‐report‐stable‐isotopic‐composition‐reference‐material‐usgs46.

[25] F. Fourel , F. Martineau , C. Lécuyer , et al., “ ^18^O/^16^O Ratio Measurements of Inorganic and Organic Materials by Elemental Analysis–Pyrolysis–Isotope Ratio Mass Spectrometry Continuous‐Flow Techniques,” Rapid Communications in Mass Spectrometry 25, no. 19 (2011): 2691–2696, 10.1002/rcm.5056. 21913245

[26] H. P. Sieper , H. J. Kupka , L. Lange , A. Roßmann , N. Tanz , and H. L. Schmidt , “Essential Methodological Improvements in the Oxygen Isotope Ratio Analysis of N‐Containing Organic Compounds,” Rapid Communications in Mass Spectrometry 24 (2010): 2849–2858, 10.1002/rcm.4714. 20857445

[27] J. du Plessis , P. d Paul , M. Kuitems , et al., “Resolving Challenges in the Development of a Protocol for δ18O Determinations on Tree‐Ring Cellulose,” In press., Isotopes in Environmental and Health Studies (2025). 10.1080/10256016.2025.2529213 40743496

[28] F. Accoe , M. Berglund , B. Geypens , and P. Taylor , “Methods to Reduce Interference Effects in Thermal Conversion Elemental Analyzer/Continuous Flow Isotope Ratio Mass Spectrometry ^18^O Measurements of Nitrogen‐Containing Compounds,” Rapid Communications in Mass Spectrometry 22, no. 14 (2008): 2280–2286, 10.1002/rcm.3609. 18561208

[29] C. E. Bopp , J. Bolotin , S. G. Pati , and T. B. Hofstetter , “Managing Argon Interference During Measurements of ^18^O/^16^O Ratios in O_2_ by Continuous‐Flow Isotope Ratio Mass Spectrometry,” Analytical and Bioanalytical Chemistry 414, no. 20 (2022): 6177–6186, 10.1007/s00216-022-04184-3. 35841416 PMC9314310

[30] S. F. Crowley , H. J. Spero , D. A. Winter , H. J. Sloane , and I. W. Croudace , “Oxygen Isotope Analysis of Carbonates in the Calcite‐Dolomite‐Magnesite Solid‐Solution by High‐Temperature Pyrolysis: Initial Results,” Rapid Communications in Mass Spectrometry 22, no. 11 (2008): 1703–1713, 10.1002/rcm.3518. 18446821

[31] T. Boschetti and P. Iacumin , “Continuous‐Flow *δ* ^18^O Measurements: New Approach to Standardization, High‐Temperature Thermodynamic and Sulfate Analysis,” Rapid Communications in Mass Spectrometry 19, no. 21 (2005): 3007–3014, 10.1002/rcm.2161. 16206239

[32] M. Gehre and G. Strauch , “High‐Temperature Elemental Analysis and Pyrolysis Techniques for Stable Isotope Analysis,” Rapid Communications in Mass Spectrometry 17, no. 13 (2003): 1497–1503, 10.1002/rcm.1076. 12820218

[33] H. Qi , M. Gröning , T. B. Coplen , et al., “Novel Silver‐Tubing Method for Quantitative Introduction of Water Into High‐Temperature Conversion Systems for Stable Hydrogen and Oxygen Isotopic Measurements,” Rapid Communications in Mass Spectrometry 24, no. 13 (2010): 1821–1827, 10.1002/rcm.4559. 20533311

[34] R. J. Gustafsson , A. Orlov , C. L. Badger , P. T. Griffiths , R. A. Cox , and R. M. Lambert , “A Comprehensive Evaluation of Water Uptake on Atmospherically Relevant Mineral Surfaces: DRIFT Spectroscopy, Thermogravimetric Analysis and Aerosol Growth Measurements,” Atmospheric Chemistry and Physics 5 (2005): 3415–3421, 10.5194/acp-5-3415-2005.

[35] R. C. Sullivan , M. J. K. Moore , M. D. Petters , S. M. Kreidenweis , G. C. Roberts , and K. A. Prather , “Timescale for Hygroscopic Conversion of Calcitemineral Particles Through Heterogeneous Reaction With Nitric Acid,” Physical Chemistry Chemical Physics 11 (2009): 7826–7837, 10.1039/B904217B. 19727489

[36] L. X. D. Chen , C. Peng , W. J. Gu , et al., “On Mineral Dust Aerosol Hygroscopicity,” Atmospheric Chemistry and Physics 20 (2020): 13611–13626, 10.5194/acp-20-13611-2020.

[37] S. F. Crowley , “Mineralogical and Chemical Composition of International Carbon and Oxygen Isotope Calibration Material NBS 19, and Reference Materials NBS 18, IAEA‐CO‐1 and IAEA‐CO‐8,” Geostandards and Geoanalytical Research 34, no. 2 (2010): 193–206, 10.1111/j.1751-908X.2010.00037.x.

[38] RM Verkouteren , DB Klinedinst , “Value Assignment and Uncertainty Estimation of Selected Light Stable Isotope Reference Materials: RMs 8543–8545, RMs 8562–8564, and RM 8566. NIST Special Publication (SP260–149),” (2004), 58 pages, last accessed January 2025, https://www.nist.gov/system/files/documents/srm/SP260‐149.pdf.

[39] A. J. Fleisher , H. Yi , A. Srivastava , O. L. Polyansky , N. F. Zobov , and J. T. Hodges , “Absolute ^13^C/^12^C Isotope Amount Ratio for Vienna Pee dee Belemnite From Infrared Absorption Spectroscopy,” Nature Physics 17, no. 8 (2021): 889–893, 10.1038/s41567-021-01226-y. PMC9982939 36873572

[40] S. Assonov , A. Fajgelj , J. F. Hélie , C. Allison , and M. Gröning , “Characterisation of new Reference Materials IAEA‐610, IAEA‐611 and IAEA‐612 Aimed at the VPDB *δ*13C Scale Realisation With Small Uncertainty,” Rapid Communications in Mass Spectrometry 35 (2021): e9014, 10.1002/rcm.9014. 33270300

