# Supplementary material for: Calcium Carbonate and Water Pyrolysis Measurements Suggest Minor Adjustment to the VPDB and VSMOW‐SLAP δ18O Scale Relation
Source: Rapid Commun Mass Spectrom. 2025 Jun 17;39(19):e10093. doi: 10.1002/rcm.10093 (PMC12171791; doi:10.1002/rcm.10093)
Supplement: Supplementary file 1 — Data S1 Supporting information. [file RCM-39-e10093-s004.docx]

SUPPORTING INFORMATION

The derivation of the conversion equations from the two different ^18^O scales based on ratios (for brevity reasons the ratio ^18^R is used as R) is provided here:

For a sample X the following applies:

$\delta{{}^{18}O}_{X (VSMOW-SLAP)}=\frac{R_{X}}{R_{VSMOW-SLAP}}-1\Longrightarrow\frac{R_{X}}{R_{VSMOW-SLAP}}=\delta{{}^{18}O}_{X (VSMOW-SLAP)}+1$ (a)

$\delta{{}^{18}O}_{X (VPDB)}=\frac{R_{X}}{R_{VPDB}}-1\Longrightarrow\frac{R_{X}}{R_{VPDB}}={\delta{}^{18}O}_{X (VPDB)}+1$ (b)

$\delta{{}^{18}O}_{VPDB (VSMOW-SLAP)}=30.92‰\Longrightarrow\frac{R_{VPDB}}{R_{VSMOW-SLAP}}=1+30.92‰=1.03092$ (c)

$\frac{R_{X}}{R_{VSMOW-SLAP}}=\frac{R_{X}}{R_{VPDB}}*\frac{R_{VPDB}}{R_{VSMOW-SLAP}}$ (d)

Filling in (a), (b) and (c) in (d) gives:

$${\delta{}^{18}O}_{X (VSMOW-SLAP)}+1=({\delta{}^{18}O}_{X (VPDB)}+1)*\left( 1.03092 \right)$$

$$\delta{{}^{18}O}_{X (VSMOW-SLAP)}=\left\{ \left( 1+ \delta{{}^{18}O}_{X (VPDB)} \right)\left( 1.03092 \right) \right\}-1$$

$$\delta{{}^{18}O}_{X (VSMOW-SLAP)}=30.92‰+\left( \delta{{}^{18}O}_{X (VPDB)}*1.03092 \right)$$

This can be rewritten as:

$\delta{{}^{18}O}_{X (VSMOW-SLAP)}= 30.92+(\left( 1+ \frac{30.92}{1000} \right)* \delta{{}^{18}O}_{X (VPDB)} ) ‰$ [1]

In analogy:

$$\delta{{}^{18}O}_{VSMOW-SLAP (VPDB)}=-29.99‰\Longrightarrow\frac{R_{VSMOW-SLAP}}{R_{VPDB}}=1-29.99‰=0.97001$$

$\delta{{}^{18}O}_{X (VPDB)}= -29.99+(\left( 1+ \frac{-29.99}{1000} \right)* \delta{{}^{18}O}_{X (VSMOW-SLAP)}) ‰$ [2]

Note that Equations 4 and 5 are in full accord with each other as:

$\frac{1}{(1+{30.92*10}^{-3})}=0.97001=(1-29.99*{10}^{-3})$

The formula for the Rayleigh fractionation for the fraction removed, that is used for fitting the data in figure 3 is:

$$\left. \delta_{m} \right.=\left( 1+{}_{0} \right)*\frac{1-\left( {N/{N_{0}})}^{\alpha} \right.}{1-\left( N/{N_{0}}) \right.}-1$$

N/N0 represents the yield fraction (N/N_0_ = 0, when yield is 100%), ${}_{0}$ refers to the original isotopic composition, α is the isotopic fractionation factor and $\delta_{m}$refers to the measured $\delta$ value of the fraction removed.

Table S1. ICP-MS results for 9 different elements in mass percentages for the calcite RMs. Based on these results, NBS-18 was not used in this study.

|  | ^23^Na (10^-2^ %) | ^24^Mg (10^-2^ %) | ^48^Ti (10^-2^ %) | ^55^Mn (10^-2^ %) | ^57^Fe (10^-2^ %) | ^80^Se  (10^-2^ %) | ^88^Sr (10^-2^ %) | ^184^Ba (10^-2^ %) | ^139^La (10^-2^ %) | Total (%) |
| --- | --- | --- | --- | --- | --- | --- | --- | --- | --- | --- |
| IAEA-603 |  | 30.07 | 1.15 |  | 1.36 |  | 1.80 |  |  | 0.34 |
| NBS-19 |  | 45.78 | 1.19 |  | 1.11 |  | 2.59 |  |  | 0.51 |
| NBS-18 | 1.46 | 46.32 | 1.08 | 25.33 | 28.30 |  | 90.57 | 7.75 | 1.26 | 2.02 |
| IAEA-610 |  |  | 1.28 |  | 0.68 |  | 1.75 |  |  | 0.04 |
| IAEA-611 | 2.22 | 0.39 | 1.65 | < 0.04 | 0.48 | < 0.04 | 0.51 | < 0.04 | < 0.04 | < 0.05 |
| IAEA-612 | 0.18 | 0.16 | 1.71 | < 0.02 | 0.48 | < 0.02 | 0.24 | < 0.02 | < 0.02 | < 0.03 |
| USGS44 |  |  | 1.09 |  | 0.94 |  |  |  |  | 0.02 |

Table S2. Typical measurement sequence pyrolysis

Benzoic Acid = BA

Blank0 = nothing

Blank1 = empty silver capsule

Blank2 = silver capsule with additives

BA, BA, Blank0, Blank0, Blank1, Blank1, Blank2, Blank2, VSMOW2 10 times, {Blank2, calcite RM} 5 times, Blank2, Blank2, sample 1 10 times, {Blank2, calcite RM} 5 times, Blank2, Blank2, sample 2 10 times, BA, BA.


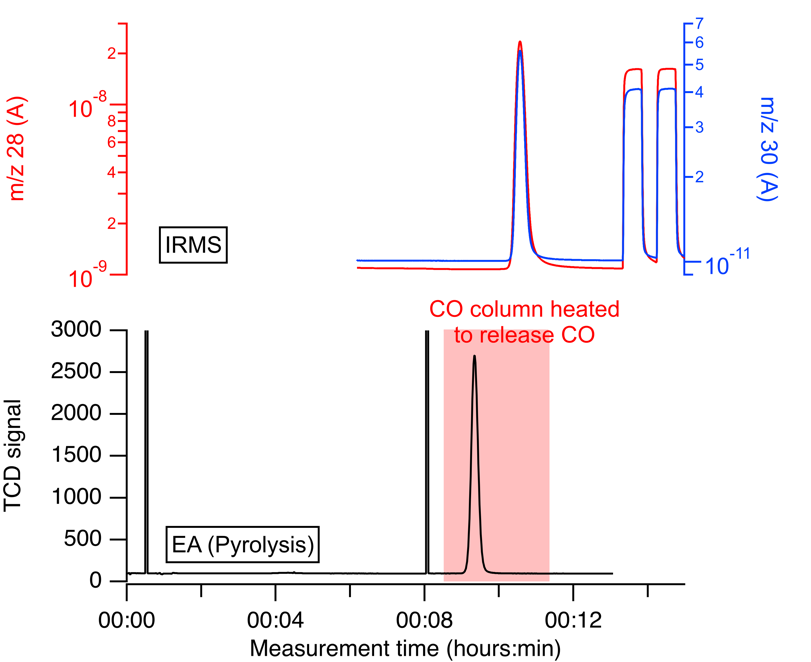


*Figure S3. The upper part of the figure is a typical IRMS mass spectrogram of CO, in red m/z 28 and in blue m/z 30. The bottom part of the figure is an EA TCD spectrum with the CO peak while the CO column was heated. The two spikes in the TCD spectrum are caused by switching valves.*


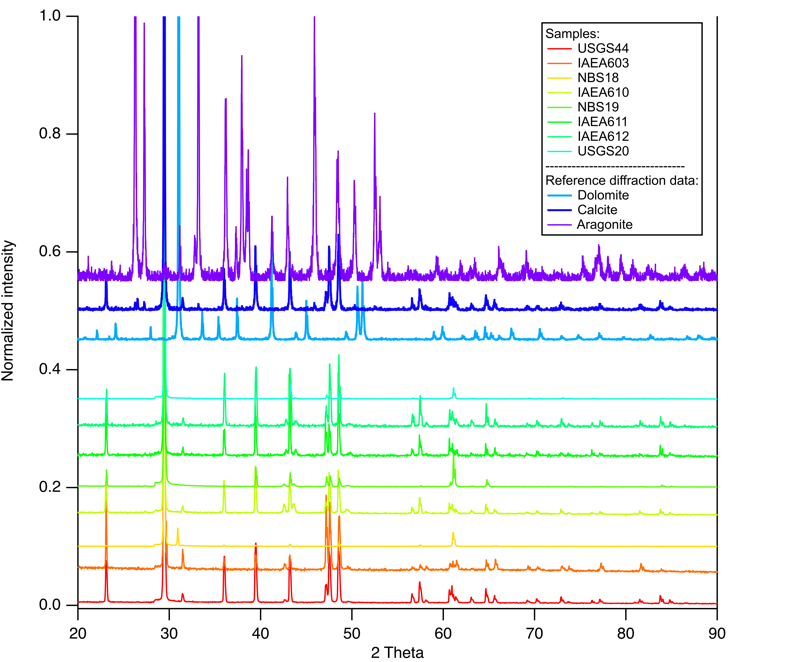


*Figure S4. PXRD spectra of all the calcium carbonate references used in this study shown together with the reference spectra of calcite, dolomite, and aragonite. For the purpose of visual clarity, a y-axis offset has been added. As is evident from the data, all materials are virtually pure calcite. The aragonite structure is fully absent, and so is dolomite, except perhaps for a small fraction in NBS 18 (which material has not been used in this study).*

Uncertainty calculation in detail

The used formulas for weighted mean $\overline{x}_{w}$, external standard deviation $\sigma_{ext}$, external error in the mean $\sigma_{m, ext}$, internal error in the mean $\sigma_{m, int}$, are listed in appendix 2 in Aerts-Bijma et al. 2021 (where the 'external error' is based on the spread of the measurements, and the 'internal error' is based on the individual uncertainties provided).

The *δ*^18^O_VMOW-SLAP_ values of Sample 1-3 were analysed using a Liquid Water Isotope Analyzer (LGR-LWIA 912-0050). Sample measurements were bracketed with local references and the international reference waters VSMOW2 and SLAP2.

Sample 1-3 (water RMs) were measured on 3 different measurement days, in every series they were measured 5 times, 10 injections each.

For each sample (1-3) the mean of 10 injections was calculated per series and its $\sigma_{m, ext}$. Then $\overline{x}_{w}$ of 5 replicants was calculated and the largest error in the mean $\sigma_{m, ext}$ or $\sigma_{m, int}$ was used as uncertainty. From the 3 different $\overline{x}_{w}$ of the 3 measurement days a final $\overline{x}_{w}$ was calculated and the largest error in the mean $\sigma_{m, ext}$ or $\sigma_{m, int}$ was used as uncertainty.

The uncertainty of the *δ*^18^O VSMOW-SLAP scale (0.03‰) was quadratically added to the last uncertainty leading to the final uncertainty for each of the samples 1-3.

Then calcium carbonate RMs and water RMs were pyrolyzed in the same batch. See the excel file *Experiment 5_uncertainty calculation example.xlsx* in DataVerseNL at <https://doi.org/10.34894/3NMVRH>. In this excel file only the relevant data for the uncertainty calculation are shown.

Each water RM was pyrolyzed 10 times, the mean, $\sigma_{ext}$, and $\sigma_{m, ext}$ were calculated (column D, E, and F). A fit line through the water RMs was drawn, with on the x-axes *δ*^18^O expected value of the water RMs (column G) and on the y-axes *δ*^18^O measured (mean) (column D). In the point of gravity of the x coordinates, the uncertainties in the slope (m2) and the intercept (m1) are independent of each other. Therefore, the equation y = m1 + m2 * (x - point of gravity) was used for the fit line. This preliminary fit line was weighted with $\sigma_{m, ext}$ (column F), so only the uncertainty in the y coordinate was taken into account.

To address the uncertainty in the x coordinate as well, this uncertainty in the x coordinate was calculated by multiplying the final uncertainty of the water RMs (determined from the optical water laser) with the slope of the fitline. (W22:W25). A new (larger) uncertainty in the y coordinate was calculated by the quadratic sum of $\sigma_{m, ext}$ (column F) and the uncertainty in the x-coordinate (W22:W25).

With the new larger uncertainty in the y coordinate a new weighted fitline was calculated and in this way both the uncertainties in the x and y coordinates were taken into account. Based on the uncertainties in the intercept (m1) and the slope (m2), the contribution of the fitline uncertainty to the calcium carbonate RMs was calculated. (column K).

The *δ*^18^O values of the calcium carbonate RMs were normalized based on the fitline through the water RMs (column J). Each calcium carbonate RM was pyrolyzed 5 times in a measurement series. The **difference** between the fitted calcium carbonate RM *δ*^18^O value (column J) with the expected *δ*^18^O values (literature values on VPDB scale converted to values on VSMOW-SLAP scale based on current recommended relation between the two ^18^O scales, column I) was calculated for each individual calcium carbonate RM measurement (column L). The assigned uncertainty (called combined uncertainty, column M) for the calcium carbonate RM in a series was the quadratic sum of $\sigma_{ext}$ (column E) of the 5 replicate calcium carbonate RM measurements and the uncertainty of the fitline (column K).

Finally 50 **differences** between the fitted calium carbonate RM *δ*^18^O value with the expected *δ*^18^O values were taken into account and 3 data points were considered as outliers, since they fall outside 1.5 times the interquartile range (IQR, range between 25^th^ and 75^th^ percentile). The $\overline{x}_{w}$ and the largest of the two $\sigma_{m, ext}$ and $\sigma_{m, int}$ were calculated of the 47 data points. In this way, we finally reached the values given in tables 4 and 5 of the main text.

Aerts-Bijma AT, Paul D, Dee MW, Palstra SWL, Meijer HAJ. An independent assessment of uncertainty for radiocarbon analysis with the new generation high-yield accelerator mass spectrometers. *Radiocarbon.* 2021;63(1):1-22. [doi:10.1017/RDC.2020.101](file:///Users/anitaaerts/surfdrive%20-%20A.T.%20Aerts-Bijma@surfdrive.surf.nl/Promotiewerk/Paper%203/First%20submission%20material%20RCM_250127/https/doi:10.1017/RDC.2020.101)
