# Supplementary material for: Calcium Carbonate and Water Pyrolysis Measurements Suggest Minor Adjustment to the VPDB and VSMOW‐SLAP δ18O Scale Relation
Source: Rapid Commun Mass Spectrom. 2025 Jun 17;39(19):e10093. doi: 10.1002/rcm.10093 (PMC12171791; doi:10.1002/rcm.10093)
Supplement: Supplementary file 2 — Table S1 ICP‐MS results for 9 different elements in mass percentages for the calcite RMs. Based on these results, NBS‐18 was not used in this study. [file RCM-39-e10093-s005.docx]

|  | ^23^Na (10^-2^ %) | ^24^Mg (10^-2^ %) | ^48^Ti (10^-2^ %) | ^55^Mn (10^-2^ %) | ^57^Fe (10^-2^ %) | ^80^Se  (10^-2^ %) | ^88^Sr (10^-2^ %) | ^184^Ba (10^-2^ %) | ^139^La (10^-2^ %) | Total (%) |
| --- | --- | --- | --- | --- | --- | --- | --- | --- | --- | --- |
| IAEA-603 |  | 30.07 | 1.15 |  | 1.36 |  | 1.80 |  |  | 0.34 |
| NBS-19 |  | 45.78 | 1.19 |  | 1.11 |  | 2.59 |  |  | 0.51 |
| NBS-18 | 1.46 | 46.32 | 1.08 | 25.33 | 28.30 |  | 90.57 | 7.75 | 1.26 | 2.02 |
| IAEA-610 |  |  | 1.28 |  | 0.68 |  | 1.75 |  |  | 0.04 |
| IAEA-611 | 2.22 | 0.39 | 1.65 | < 0.04 | 0.48 | < 0.04 | 0.51 | < 0.04 | < 0.04 | < 0.05 |
| IAEA-612 | 0.18 | 0.16 | 1.71 | < 0.02 | 0.48 | < 0.02 | 0.24 | < 0.02 | < 0.02 | < 0.03 |
| USGS44 |  |  | 1.09 |  | 0.94 |  |  |  |  | 0.02 |
