# Supplementary material for: Calcium Carbonate and Water Pyrolysis Measurements Suggest Minor Adjustment to the VPDB and VSMOW‐SLAP δ18O Scale Relation
Source: Rapid Commun Mass Spectrom. 2025 Jun 17;39(19):e10093. doi: 10.1002/rcm.10093 (PMC12171791; doi:10.1002/rcm.10093)
Supplement: Supplementary file 3 — Table S2 Typical measurement sequence pyrolysis. [file RCM-39-e10093-s002.docx]

Benzoic Acid = BA

Blank0 = nothing

Blank1 = empty silver capsule

Blank2 = silver capsule with additives

BA, BA, Blank0, Blank0, Blank1, Blank1, Blank2, Blank2, VSMOW2 10 times, {Blank2, calcite RM} 5 times, Blank2, Blank2, sample 1 10 times, {Blank2, calcite RM} 5 times, Blank2, Blank2, sample 2 10 times, BA, BA.
