# Supplementary figures and images for: Calcium Carbonate and Water Pyrolysis Measurements Suggest Minor Adjustment to the VPDB and VSMOW‐SLAP δ18O Scale Relation
Source: Rapid Commun Mass Spectrom. 2025 Jun 17;39(19):e10093. doi: 10.1002/rcm.10093 (PMC12171791; doi:10.1002/rcm.10093)

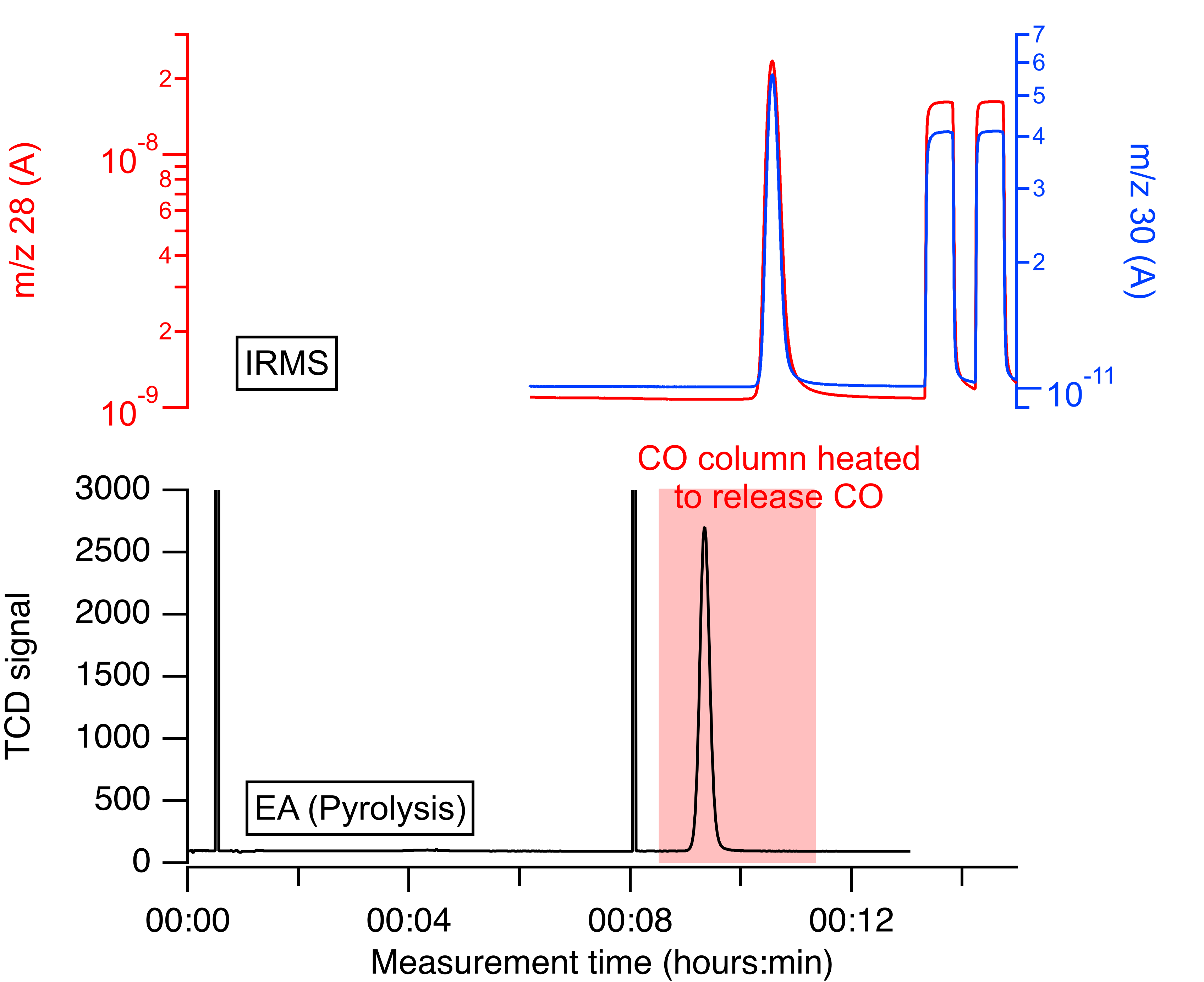

Supplement: Supplementary file 4 — Figure S3 The upper part of the figure is a typical isotope ratio mass spectrometer mass spectrogram of CO, in red m/z 28 and in blue m/z 30. The bottom part of the figure is an EA TCD spectrum with the CO peak while the CO column was heated. The two spikes in the TCD spectrum are caused by switching valves. [file RCM-39-e10093-s003.png]

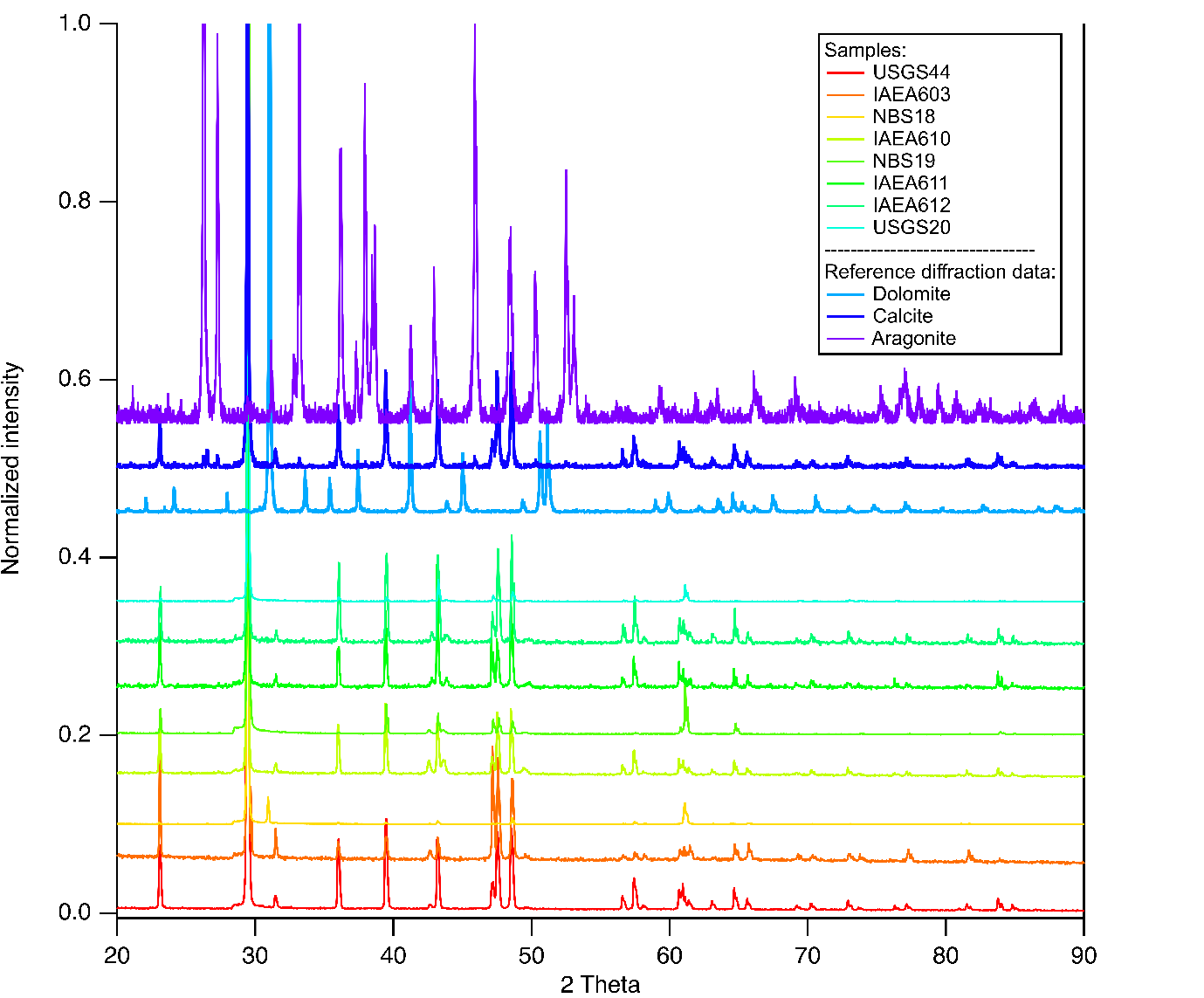

Supplement: Supplementary file 5 — Figure S4 PXRD spectra of all the calcium carbonate references used in this study shown together with the reference spectra of calcite, dolomite, and aragonite. For the purpose of visual clarity, a y‐axis offset has been added. As is evident from the data, all materials are virtually pure calcite. The aragonite structure is fully absent, and so is dolomite, except perhaps for a small fraction in NBS 18 (which material has not been used in this study). [file RCM-39-e10093-s001.docx]
